# (R)Evolution of Refrigerants

**DOI:** 10.1021/acs.jced.0c00338

**Published:** 2020

**Authors:** Mark O. McLinden, Marcia L. Huber

**Affiliations:** Applied Chemicals and Materials Division, National Institute of Standards and Technology, Boulder, Colorado 80305, United States; Applied Chemicals and Materials Division, National Institute of Standards and Technology, Boulder, Colorado 80305, United States

## Abstract

As we enter the “fourth generation” of refrigerants, we consider the evolution of refrigerant molecules, the ever-changing constraints and regulations that have driven the need to consider new molecules, and the advancements in the tools and property models used to identify new molecules and design equipment using them. These separate aspects are intimately intertwined and have been in more-or-less continuous development since the earliest days of mechanical refrigeration, even if sometimes out-of-sight of the mainstream refrigeration industry. We highlight three separate, comprehensive searches for new refrigerants–in the 1920s, the 1980s, and the 2010s–that sometimes identified new molecules, but more often, validated alternatives already under consideration. A recurrent theme is that there is little that is truly new. Most of the “new” refrigerants, from R-12 in the 1930s to R-1234yf in the early 2000s, were reported in the chemical literature decades before they were considered as refrigerants. The search for new refrigerants continued through the 1990s even as the hydrofluorocarbons (HFCs) were becoming the dominant refrigerants in commercial use. This included a return to several long-known natural refrigerants. Finally, we review the evolution of the NIST REFPROP database for the calculation of refrigerant properties.

## Graphical Abstract

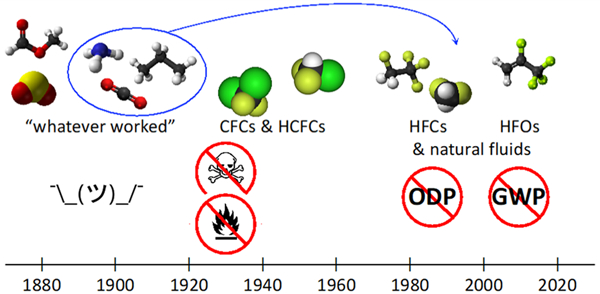

## 1. INTRODUCTION

Refrigeration was named one of the 20 most significant engineering achievements of the 20th Century in a recent review, ranking alongside computers, spacecraft, and the Internet.^1^ Nearly 20% of energy consumption worldwide is due to the demand for refrigeration and air conditioning.^2^ Refrigerants are the vital working fluids at the heart of the vapor-compression cycle, moving heat from a lower to higher temperature.

In the present work we trace the evolution of refrigerants from the early days of mechanical refrigeration to the present. We consider the evolution of the molecules themselves, making use of the refrigerant “generations” defined by Calm,^3^ as well as the evolution of the constraints on the characteristics required of refrigerants, which have largely driven the need to find and develop new refrigerants. We also consider the evolving tools and models used to represent the properties of refrigerants and their mixtures, with a focus on the NIST REFPROP^4^ database. In some cases, these developments were revolutionary, thus, the “(R)” in the title. We also note examples where previously known molecules have been applied (or reapplied) to meet new constraints; in other words, sometimes, the choice of refrigerants has “revolved,” in the sense of “circling back.” Our title is, thus, a play on words.

## 2. EVOLVING CONSTRAINTS AND REFRIGERANT “GENERATIONS”

Although the search for the ideal refrigerant may seem to be a modern endeavor spurred by the need to replace the chlorofluorocarbons (CFCs) in the 1980s or, more recently, to find low-global warming potential (GWP) fluids, in fact, the search for the “ideal” refrigerant dates back to virtually the beginning of mechanical refrigeration. An 1847 essay posited that “… mankind has been incessantly in quest of refrigeratives.”^5^ While that early work focused on means of producing cold known since ancient times (e.g., the storage of winter ice or the evaporation of water from porous earthenware vessels) it also declared that “liquid carbonic acid, takes the highest rank as a frigorific agent” and noted that a “Mr Addams of Kensington actually manufactures this curious liquid as an article of commerce.”

### 2.1. Early Refrigerants.

Calm^3,6^ described the development of refrigerants in terms of four “generations,” and he characterized the first generation (spanning from the beginning of artificial refrigeration up to the 1930s) as “whatever worked.” Indeed, the main constraints on a refrigerant in this era were simply that it be available and that it worked in the equipment of the time. Nevertheless, some of the refrigerants identified in these early years included fluids with thermodynamic characteristics that make them excellent refrigerants to this day (e.g., ammonia, propane, CO_2_) as well as fluids, such as sulfur dioxide, which had good thermodynamic characteristics but were highly toxic.

A systematic search in 1924 for suitable refrigerants, which included an analysis of thermodynamic efficiency and consideration of chemical stability,^7^ settled on dichloroethylene (i.e., *trans*-1,2-dichloroethene) for use in centrifugal compressors. This fluid is now called R-1130(E) and is a component of the azeotropic blend with R-1336mzz(Z) known as R-514A,^8^ which has been recently commercialized as a replacement for R-123 in centrifugal chillers. Seemingly, nothing is new.

**Figure 1.**
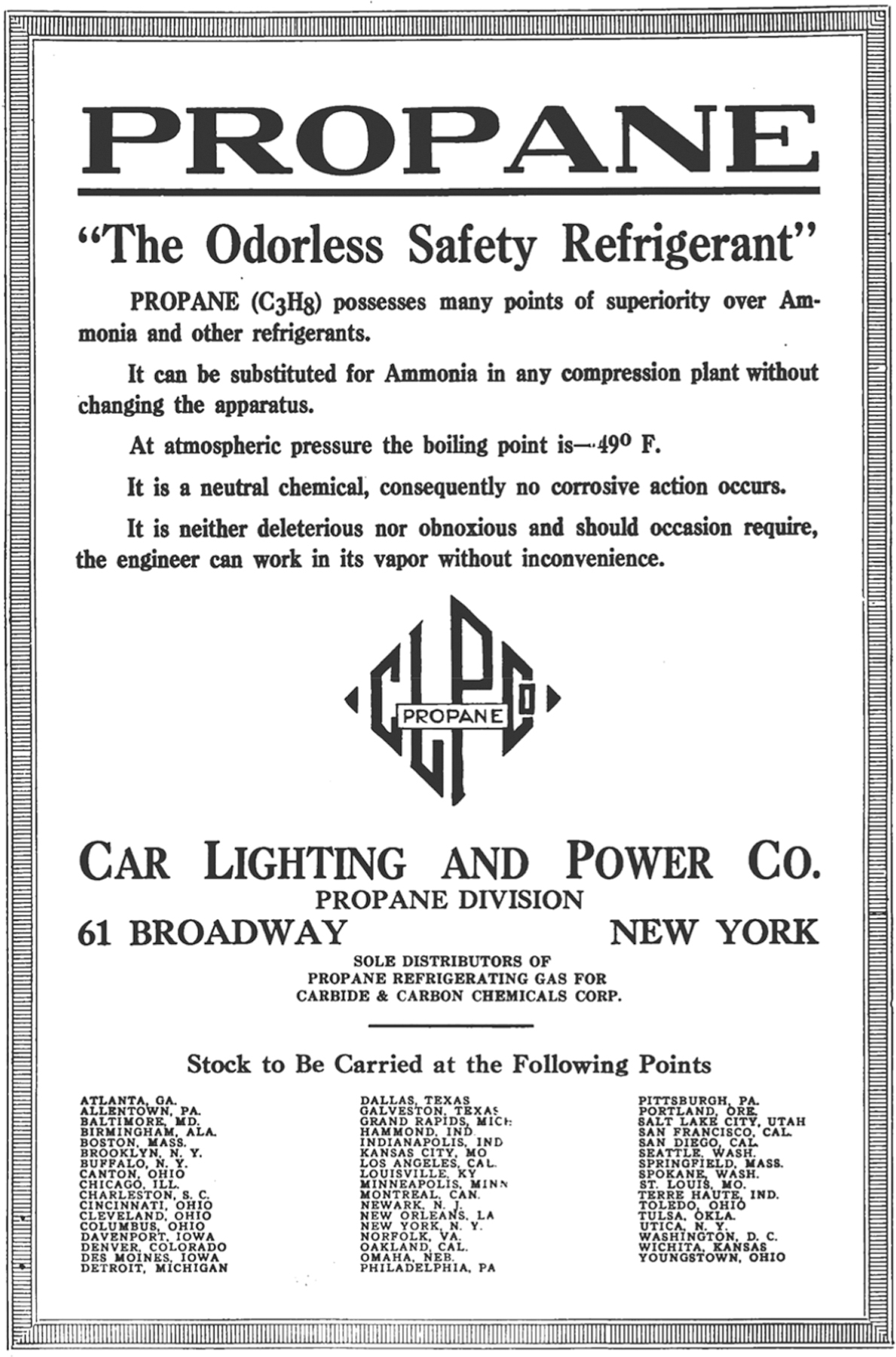
Advertisement for propane appearing in the December 1922 issue of *Ice and Refrigeration* magazine.

Refrigeration in this period was primarily applied in industrial settings to the production of ice. Toward the end of this era, the first household refrigerators began to be produced. The “Monitor Top” refrigerator, introduced by General Electric^9^ in 1927, was considered to be the first affordable refrigerator.^10^ These early units used sulfur dioxide, methyl formate, ammonia, or methyl chloride as the refrigerant–all toxic and flammable fluids. The contemporary view of some of the refrigerants would now be considered quite odd. In 1922, propane was advertised as the “odorless safety refrigerant”^11^ (see Figure 1) in contrast to the pungent odor of ammonia or sulfur dioxide. Given that the other common refrigerants of the time were also flammable, the low toxicity of propane was apparently sufficient to inspire the label of “safety.” The hazard of sulfur dioxide, on the other hand, was considered manageable,^12^ because it is “self warning”, i.e., its odor can be detected well below hazardous levels. An article in the June 1919 issue of *Popular Science* touted a household refrigerator that “does away with the iceman” but made no mention of its use of liquid sulfur dioxide.^13^ Its current recommended exposure limit of 2 ppm (parts per million by volume) averaged over a 8 h work shift^14^ is today considered “highly toxic.”

### 2.2. Introduction of Halogenated Refrigerants.

Given the refrigerants in use in the 1920s, Charles Kettering, head of research at General Motors (which was the parent company to Frigidaire, an early maker of domestic refrigerators), declared to Thomas Midgely that “the refrigeration industry needs a new refrigerant if they ever expect to get anywhere.”^15^ The desired properties were “a boiling point between 0 and −40 °C, stability, nontoxicity, and nonflammability.”

**Figure 2.**
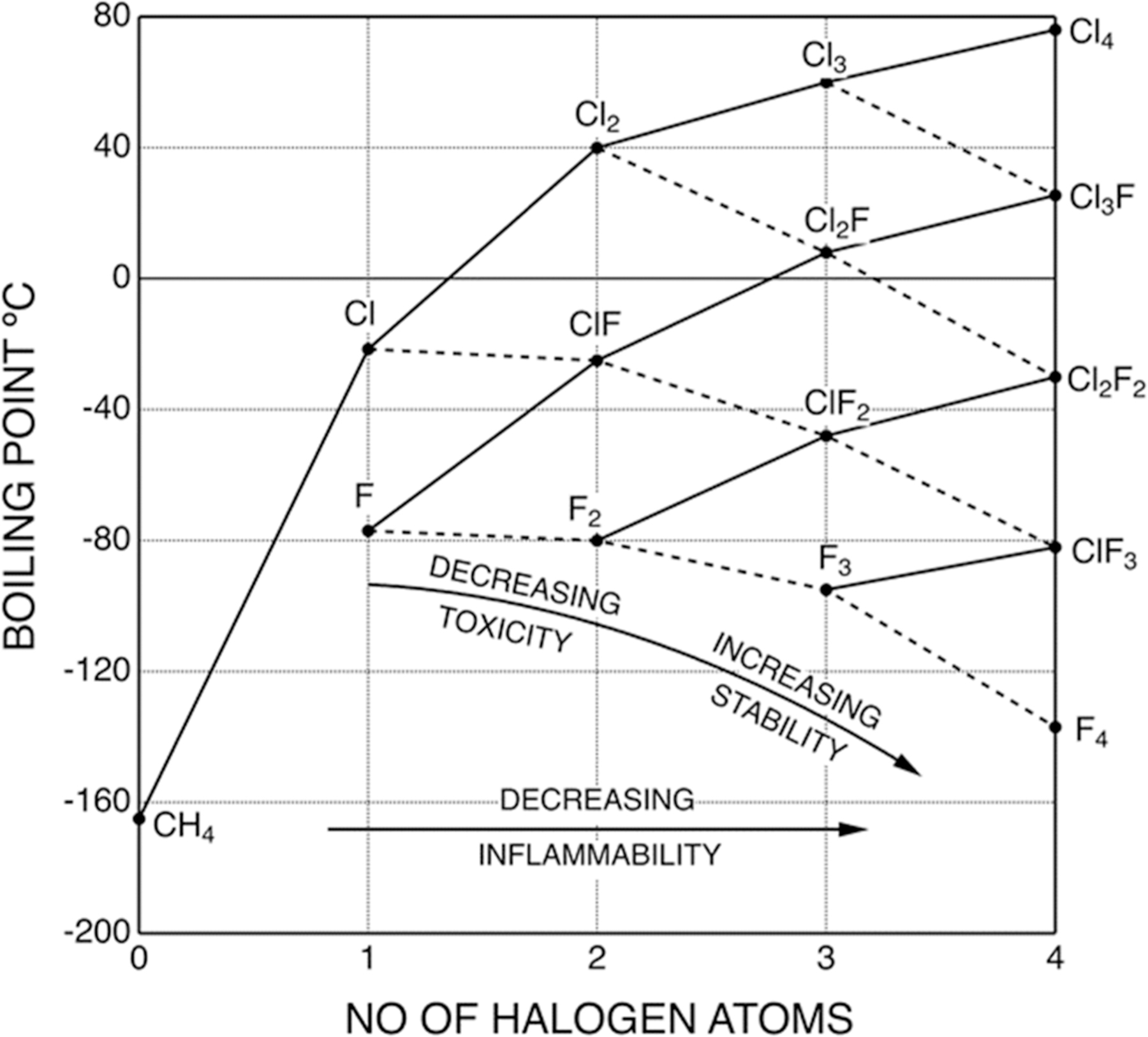
Systematic examination of the fluorochloro derivatives of methane; redrawn from Midgley and Henne^16^ for legibility. Note that the values of normal boiling point are those plotted by Midgley and Henne and may differ from currently accepted values. (Only halogens are indicated; carbon and hydrogen are understood.).

Midgley, together with Albert Henne and Robert MacNeary, set out to find a better refrigerant. They examined the periodic table of the elements and noticed patterns. Many of the elements were metals and were excluded from consideration because they formed nonvolatile compounds when combined with other elements. The noble gases were excluded due to their extremely low boiling points. Among the nonmetallic elements, substances that primarily formed toxic or unstable compounds were eliminated. This left only eight elements: carbon, nitrogen, oxygen, sulfur, hydrogen, fluorine, chlorine, and bromine. Midgley and his colleagues also noticed general trends of flammability and toxicity tending to decrease as one moved from left to right and from bottom to top in the periodic table. This led them to look at compounds of chlorinated and fluorinated hydrocarbons, and they noted how variations in the degree of chlorination and fluorination influenced the boiling point, flammability, and toxicity.^16^ They identified dichlorofluoromethane (R-21) as a promising candidate, synthesized a small quantity of it, and found it to be of low toxicity. Investigation of other chlorofluorocarbons followed, and commercial production of dichlorodifluoromethane (R-12) began in 1931 followed by trichlorofluoro-methane (R-11) in 1932.^3^ Figure 2 illustrates the systematic nature of this investigation.

Thus, began the “second generation” of refrigerants for “safety and durability.”^3^ They were often collectively called “Freons”, after the registered trademark of one of the major refrigerant manufacturers.

The development of R-12 represents perhaps one of the few examples of synthesizing a new molecule specifically for use as a refrigerant and was truly a revolutionary development. (As discussed below, most subsequent “new” refrigerants had been previously identified in the chemical or patent literature for other purposes.) Although dichlorodifluoromethane was reported in the chemical literature as early as 1907,^17^ Midgely was apparently unaware of this, and there are no other citations to dichlorodifluoromethane listed in the SciFinder database between 1907 and the seminal 1930 paper by Midgley and Henne.^16^

Through the 1930s, 1940s, and 1950s additional CFCs and HCFCs (hydrochlorofluorocarbons) were developed and commercialized. Significant among these was R-22 (an HCFC), which was first commercialized in 1936, although it was not until the late 1950s that it saw widespread use in air-conditioning systems.^18^ Also in the 1950s, mixtures of CFCs and HCFCs came into use as a way to increase volumetric refrigeration capacity, lower compression ratios, or otherwise tailor the properties of the refrigerants to a particular application.^19^ These were azeotropic mixtures and, thus, could be handled in the same way as single-component fluids.

A wider range of chemicals continued to be considered. A 1944 study by Markham^20^ carried out at the York Research Laboratory (now part of Johnson Controls International) considered a total of 91 chemicals with normal boiling points ranging from nitrogen (*T*_b_ = −195.8 °C) to water (*T*_b_ = 100 °C) that had been “patented, used, or suggested in the literature as refrigerants.” They ranged from well-known chemicals to those for which only the boiling point temperature was reported. Included were a number of HFCs (e.g., R-32, R-125, and R-134) and HCFCs (e.g., R-123) many years before they were commercialized. Also included were several compounds now known to be highly toxic (e.g., BF_3_ and AsF_5_) as well as hydrocarbons and several ethers.

The CFCs and HCFCs came to dominate most refrigeration and air-conditioning applications, and this period lasted roughly 60 years, into the 1990s.^3^ Ammonia in industrial-scale refrigeration applications, such as cold stores and breweries, was the main holdover from the early refrigerants.

### 2.3. Evolving Constraints and Safety Standards.

As mentioned in the previous two sections, the constraints on early refrigerants were rather basic. But, as the synthetic refrigerants were developed and commercialized, and nonflammable refrigerants of low toxicity became available, previously “desirable” characteristics became mandatory constraints under safety codes and standards. (“Standards” define best practices but are usually voluntary. “Codes” define required practices and may be enforced by various government agencies. Codes often incorporate the practices defined in standards.)

The most widely recognized safety standard in the United States is *ANSI/ASHRAE Standard 15*−*2019 Safety Standard for Refrigeration Systems*.^21^ The analogous international standard is *ISO 5149: Mechanical refrigerating systems used for cooling and heating*−*Safety requirements*.^22^ Faust^23^ describes the evolution of the ASHRAE standard. The first edition was published in 1919 and focused on ammonia systems in cold storage and ice-making plants. It was very basic, with classes of equipment defined by the refrigeration capacity of the system; it specified, for example, the installation of systems in machinery rooms, the use of safety relief valves, and maximum operating pressures. The classification of systems was changed in 1922 to one based on the total charge of refrigerant. It was changed again in 1929 to reflect the application (industrial, commercial, apartments, etc.). A further change in 1939 to classify systems by class of building occupancy remains in place to this day. This reflects the hazard presented to building occupants, ranging from “industrial” where only authorized and trained persons are present, to residential, to “public assembly” where large groups of people may be present, to institutional, e.g., hospitals and prisons, where mobility may be limited.

The 1939 edition also introduced a classification system for the refrigerants themselves. The “Group 1” refrigerants (e.g., many of the CFCs) were nonflammable and of low toxicity, as defined by the effects of a short-term exposure on guinea pigs. “Group 2” refrigerants (e.g., ammonia) “may or may not be flammable” and were “toxic to the degree that they will produce death or serious injury to guinea pigs during a 2 h exposure… at a concentration of 2−1/2 percent.”^24^ “Group 3” refrigerants (e.g., the hydrocarbons) were flammable, but of low toxicity. This simplified adding new refrigerants to the standard.

The classification of refrigerants was spun off to a new standard first published in 1957. The current version is *ANSI/ASHRAE Standard 34–2019 Designation and Safety Classification of Refrigerants*.^21^ Groups 1, 2, and 3 of the 1939 mechanical safety standard were retained through the 1978 edition.^24^ The 1989 edition^25^ subdivided the (flammable) Group 3 into “3a” and “3b” based on the lower explosion limit (now referred to as lower flammability limit) and heat of combustion. It also introduced a new Group 4 for mixtures of Group 1 and Group 3 refrigerants to accommodate the interest in such mixtures at the time. That classification scheme was increasingly seen as illogical: Standard 15 placed greater restrictions on a Group 3 (flammable, but nontoxic) refrigerant than a Group 2 (flammable and toxic) refrigerant.

The 1992 edition of Standard 34^26^ introduced a new classification scheme based on separate toxicity and flammability criteria. The toxicity classes became “A” (“lower toxicity”) or “B” (“higher toxicity”) based on the “TLV−TWA” (threshold limit value−time-weighted average), an indicator of permissible long-term exposure in an industrial setting. This was a much more comprehensive indicator of toxicity than the earlier ranking based on a single test with guinea pigs. The current standard references an “OEL” or occupational exposure limit, which is similar to the TLV−TWA, but can be based on a number of different indices from various sources.

**Figure 3.**
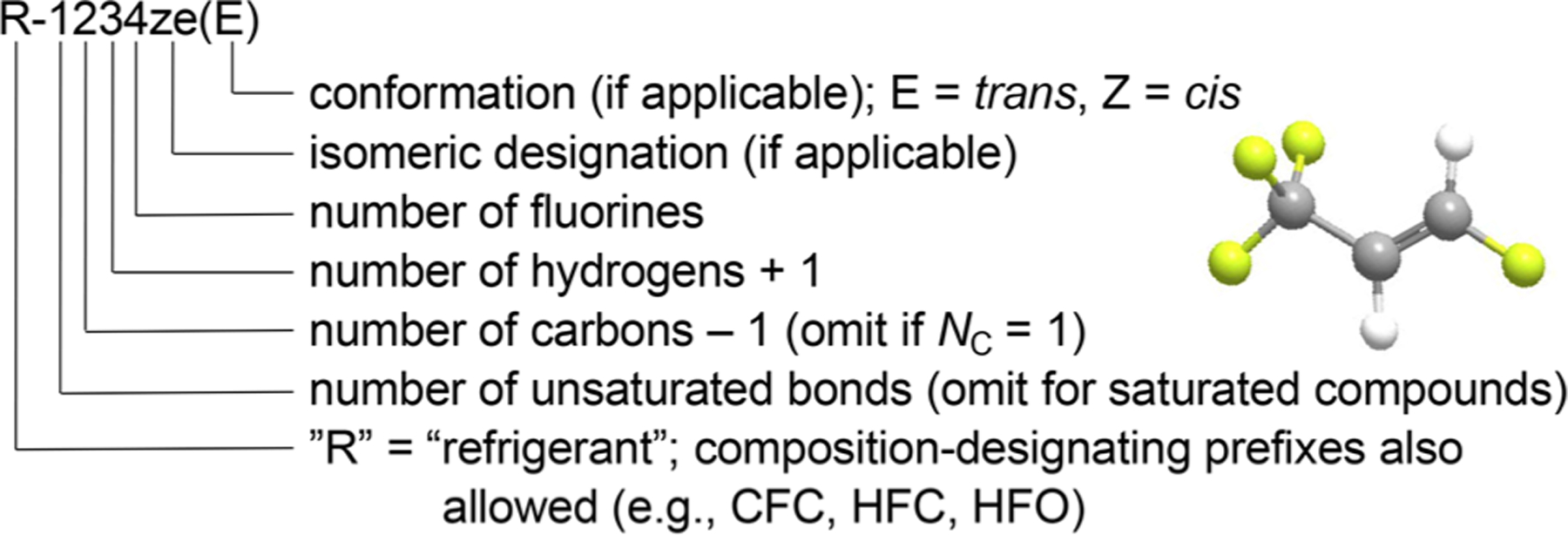
Refrigerant nomenclature system of ASHRAE Standard 34,^8^ taking R-1234ze(E), *trans*-1,1,1,3-tetrafluoropropene, as an example.

Refrigerants in flammability class “1” show “no flame propagation.” Class “2” refrigerants were of “lower flammability” as defined by a lower flammability limit (LFL) of more than 0.10 kg/m^3^ *and* a heat of combustion less than 19 MJ*·* kg^−1^. Class “3” refrigerants were of “higher flammability” as defined by a lower flammability limit of less than 0.10 kg*·*m^−3^ *or* a heat of combustion greater than 19 MJ*·*kg^−1^. These limits were selected to put ammonia into Class 2, but near the Class 2/3 boundary, with the logic that it was the archetypical “Group 2” refrigerant that enjoyed fewer restrictions than the Group 3 refrigerants. The LFL is defined by testing in accordance with the ASTM E681–85 method.^27^ The 2010 edition^28^ added a “2L” subdivision of Class 2 for refrigerants with a burning velocity less than 10 cm*·*s^−1^. The 2019 edition^8^ defined “2L” as a separate class and assigned new descriptors to the classes; Class 2L is now described as “lower flammability” and Class 2 as “flammable.” Thus, there are now eight possible classifications: A1, A2L, A2, A3, B1, B2L, B2, and B3. ASHRAE Standard 34 also defines a standard nomenclature for refrigerants. This is illustrated in Figure 3.

The current version of ASHRAE Standard 15 does not generally mandate the use of a particular class of refrigerant in a particular class of occupancy. Rather, the quantity of allowable refrigerant charge is limited based on the hazard of each particular refrigerant, such that the release of the refrigerant charge (resulting from equipment failure or other means) into an occupied space does not exceed a concentration that would present an immediate toxicity hazard or would reach 25% of the LFL. This “refrigerant concentration limit” or RCL is defined in ASHRAE Standard 34 and varies widely; for example, it is 50 000 ppm (parts per million by volume) for R-134a and 320 ppm for ammonia. This is of consequence mainly for “high-probability systems” where leakage from the system will enter the occupied space. “Low-probability systems” employing, for example, a secondary coolant loop or systems in separate machinery rooms, are subject to lesser restrictions. The net effect is that only class A1 refrigerants can be used in many applications, because it is impractical with current designs to, for example, have a residential air-conditioning system with a sufficiently small charge to allow the use of a toxic or flammable refrigerant. With the 2019 edition of Standard 15, however, provisions were added to allow a broader use of Class 2L refrigerants compared to the earlier provisions that applied to all of Class 2.

The concept of the RCL was introduced in 2007. This represented the culmination of a trend in the standards from the specification of specific refrigerants allowable in particular applications to more general specifications written in terms of quantifiable hazards.

### 2.4. Response to Ozone Depletion.

By the 1980s the CFCs and HCFCs were used not only as refrigerants but as foam-blowing agents, cleaning solvents, and aerosol propellants for personal care products. They were everywhere. In 1973 Lovelock et al.^29^ reported that nearly all of the R-11 and R-12 that had ever been produced was still in the atmosphere. Their interest was in using the CFCs as tracer gases for atmospheric studies. They stated that “The presence of these compounds constitutes no conceivable hazard” and “CCl_3_F [R-11] … does not disturb the environment.” The next year Molina and Rowland^30^ hypothesized that the sink for the CFCs was in the stratosphere where they would be dissociated by ultraviolet radiation, releasing atomic chlorine that would destroy ozone. Following corroborating studies, the use of CFCs as aerosol propellants was banned in 1978 in the United States. By 1982 many further studies had been carried out, and a summary by the U.S. National Academy of Sciences^31^ concluded that continued production of R-11 and R-12 at 1977 levels would result in an eventual reduction in global ozone of 5 to 7%. The issue of ozone depletion faded from public consciousness, although research on the topic continued, as did the use of the CFCs as refrigerants.

The Vienna Convention for the Protection of the Ozone Layer^32^ was approved in 1985; this treaty provided for the sharing of research on ozone and established a framework for regulating the production of ozone-depleting substances. Shortly after, Farman et al.^33^ discovered the “ozone hole” over the Antarctic. Ozone exists at relatively high concentrations in the stratosphere; the “ozone hole” is not really a hole where no ozone is present, but rather an area of reduced ozone concentration in the stratosphere over the southern polar region. It is defined as the region over Antarctica with a total ozone concentration of 220 Dobson units or lower.^34^ The following year Solomon et al.^35^ reported balloonsonde data collected in the Antarctic spring that demonstrated that chlorine chemistry occurring on the surfaces of polar stratospheric clouds were responsible for the ozone hole and that the ClO (chlorine monoxide) behind these reactions originated from the CFCs.

These discoveries renewed the urgency to reduce the use of CFCs and led to the adoption of the Montreal Protocol on Substances that Deplete the Ozone Layer^36^ in 1987. The original Montreal Protocol called only for a 50% reduction in the production of five of the most-common CFCs (including R-11 and R-12) as well as three Halons (used as fire-extinguishing agents), and some in the refrigeration industry felt that the remaining 50% of CFC production should be reserved for refrigerant usage. But, subsequent amendments to the Montreal Protocol mandated the complete phase-out of the CFCs as well as several HCFCs. Thus, began the “third generation” of refrigerants, characterized by Calm^3^ as “ozone protection.”

With the ozone hole and Montreal Protocol the refrigeration industry was suddenly faced with finding replacements for the CFCs. R-134a (1,1,1,2-tetrafluoroethane) and R-123 (2,2-dichloro-1,1,1-trifluoroethane) had been identified as possible CFC replacements by one of the refrigerant manufacturers^37^ as early as 1977. But these fluids were known well before that. Indeed, R-134a was mentioned as a possible refrigerant in a 1959 patent,^38^ and it was mentioned in the chemical literature in 1936.^39^ The first investigation of R-123 was as an anesthetic in 1946 by Robbins,^40^ who used material prepared by McBee et al.^41^

#### 2.4.1. National Bureau of Standards (NBS) Study of Alternatives.

But were these the best fluids? To answer this, first, we need to know the characteristics required of a refrigerant. McLinden and Didion^42^ laid out a list of requirements for a working fluid in a vapor compression refrigeration cycle, listed in Table 1. They argued that stability within the refrigeration system was the most important requirement, because all other properties would be moot if the material reacted or degraded in use. The next most important requirements related to health, safety, and environmental aspects; for this last category they stated that “a refrigerant should not contribute to ozone depletion, low level smog formation, or greenhouse warming.” Thermophysical properties were important for maximizing the performance of a refrigeration system, and other “miscellaneous” properties were important for practical reasons. They concluded that trade-offs were inevitable.

**Figure 4.**
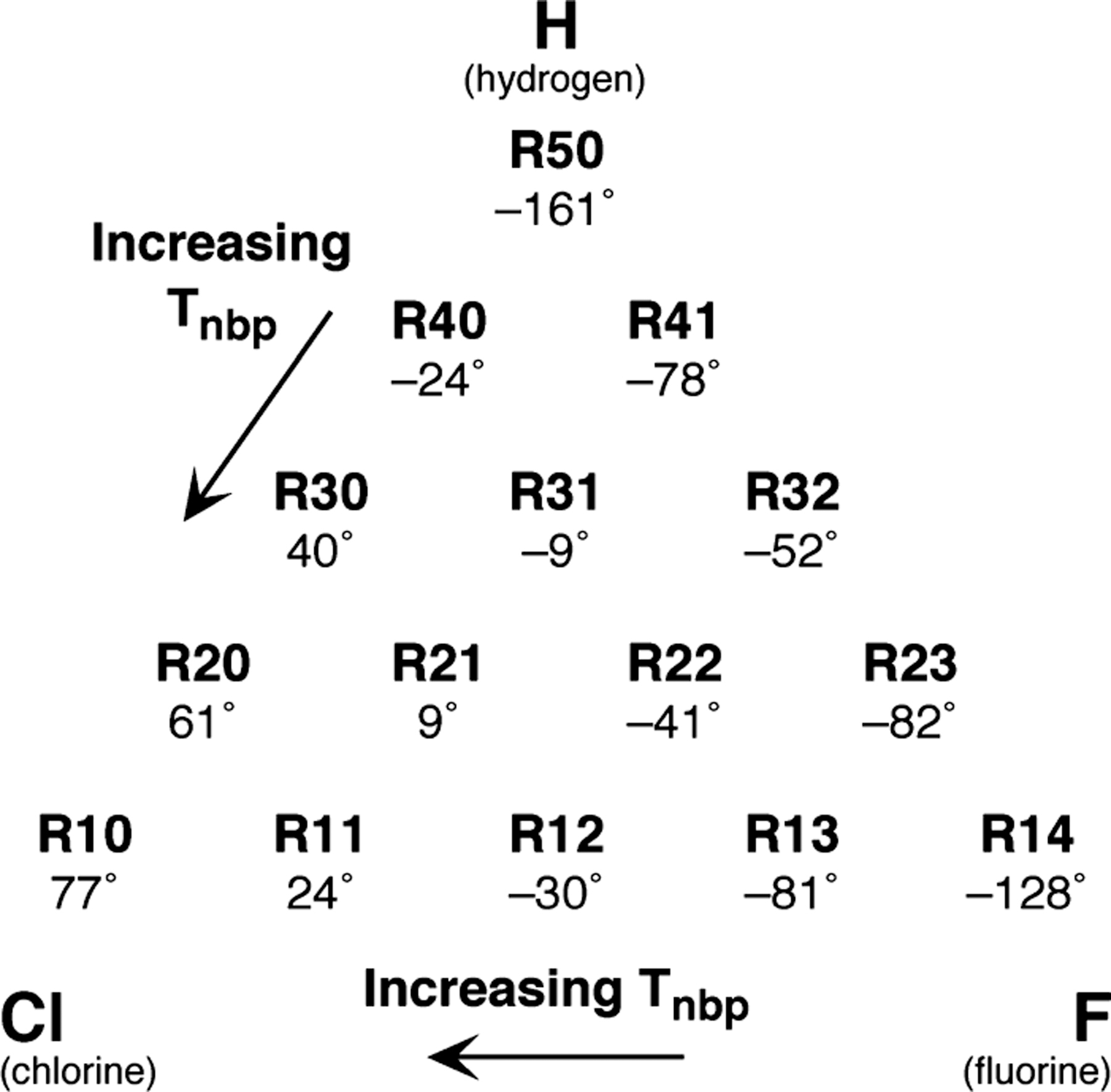
Patterns in the normal boiling point temperature (°C) among the methane-based (one-carbon) halocarbon refrigerants; Figure adapted with permission from ref 42. Copyright 1987 ASHRAE, www.ashrae.org.

**Figure 5.**
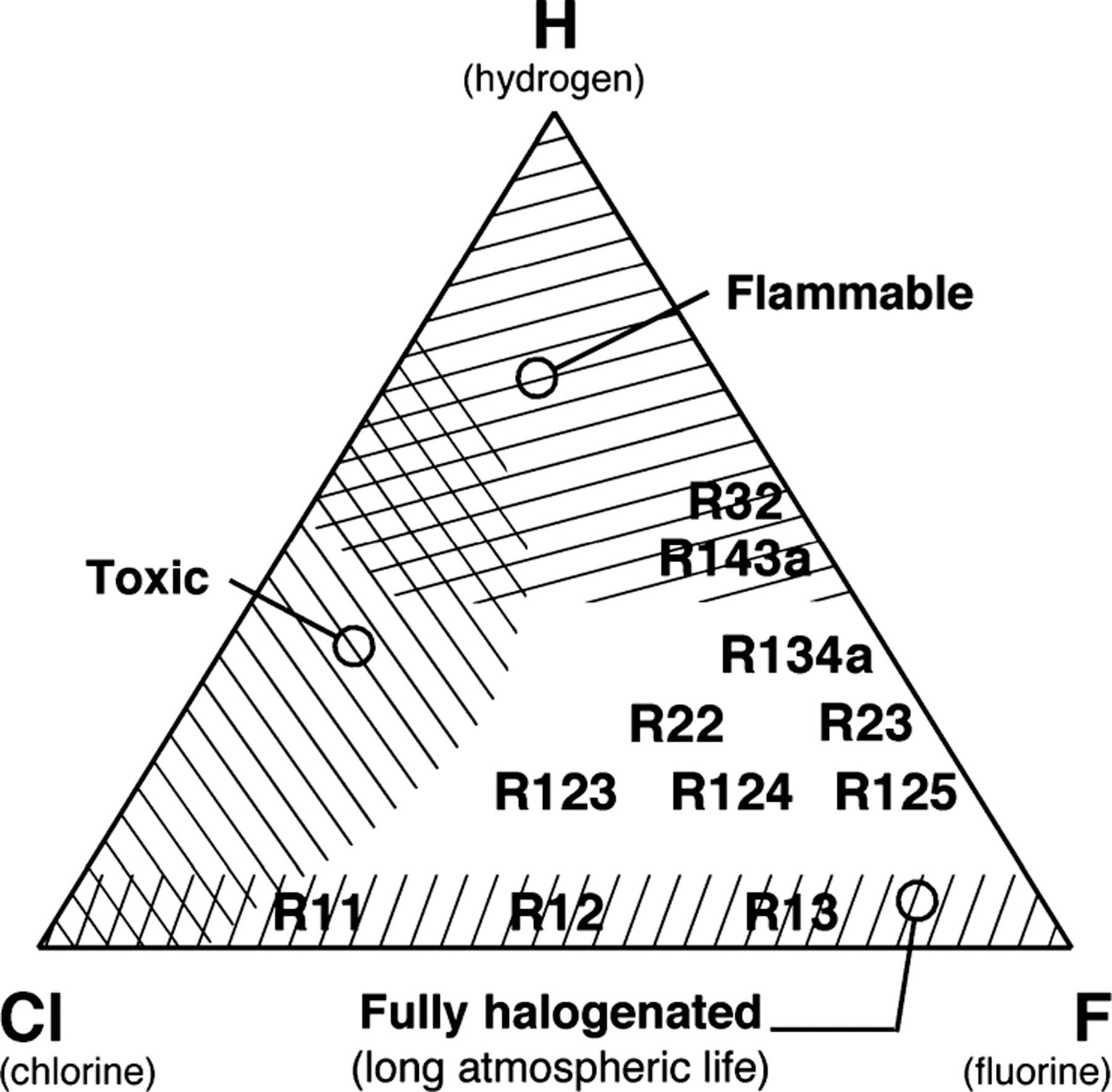
Summary of refrigerant characteristics; most of the “new” refrigerants identified to replace the CFCs were clustered in the region that is neither toxic nor flammable nor fully halogenated. Figure adapted with permission from ref 42. Copyright 1987 ASHRAE, www.ashrae.org.

McLinden and Didion^42^ went on to consider possible candidates by three separate lines of analysis. A database search that applied simple thermal criteria to a list of 860 industrial chemicals revealed 51 candidates, including some halocarbons that were both nonflammable and of low toxicity. They also noted that 49 of the 51 comprised the same eight elements considered by Midgley^15^ (i.e., C, N, O, S, H, F, Cl, Br). They laid out chemical and thermodynamic arguments that pointed to one- and two-carbon HFCs and HCFCs as possessing the most promising combination of properties. (They dismissed the halogenated olefins because of their lower stability.) Finally, they examined trends for boiling point, flammability, toxicity, and atmospheric lifetime (as a proxy for both ozone depletion potential (ODP) and global warming potential (GWP)) among the halocarbons in terms of triangular diagrams with the base hydrocarbon (methane or ethane) at the apex, with increasing halogenation as one moved lower in the diagram. Figure 4 shows an example diagram for the boiling point of the methane derivatives, and Figure 5 shows a summary of the trade-offs for both the methane and ethane derivatives.

The 1987 analysis of McLinden and Didion^42^ indicated that R-134a was indeed a good replacement for R-12 in automotive air-conditioning and that R-123 was a good replacement for R-11 in systems with low-pressure centrifugal compressors. This gave confidence to the refrigeration industry as they phased out these CFCs. As the Montreal Protocol was amended over time to also phase out the HCFCs, including R-22, the “acceptable” area in the middle of Figure 5 disappeared, leaving only R-134a and R-125 as nonflammable, low-toxicity, zero-ODP fluids. (R-23, with a normal boiling point of −82.0 °C, is too volatile to serve as an R-22 replacement.) Still, a mixture of (flammable) R-32 with R-125, known as R-410A^8^, proved to have good properties and has come to dominate small air-conditioning and heat-pumping systems that previously used R-22.^43^

Although McLinden and Didion^42^ posited that trade-offs were inevitable, entirely satisfactory replacement refrigerants (based on the HFCs) were identified and implemented. To be sure, there were disruptions, and extensive research was required to identify lubricants, elastomers, hose materials, etc. that would be compatible with the new refrigerants, as well as research to determine the thermophysical properties of the replacements. But, in the end, no trade-offs in safety (toxicity or flammability) were required, and the new refrigerants often proved to be more energy efficient than the old CFCs once systems were optimized for the properties of the new fluids.

#### 2.4.2. Refrigerant Blends.

The NBS work on replacement refrigerants had its origins in a program initiated by Didion^44^ in 1981 to study refrigerant blends as a means to increase the efficiency of heat pumps. The program investigated the cycle itself, impacts on heat transfer, and the effects of refrigerant properties on heat pump performance. In 1987 the NBS group realized that they were in a unique position of considering the effects of refrigerant properties on the performance of a refrigeration cycle at a time when virtually all refrigerators used R-12, all air-conditioners used R-22, etc. The initial study of CFC replacements described in the previous section considered pure-component refrigerants, but the subsequent mandate to phase out R-22 presented a real challenge: there was no suitable pure fluid that could match its properties. Industry responded by developing refrigerant mixtures that were to replace R-22: R-407C (a blend of R-32/125/134a), which closely matched the properties of R-22 and R-410A (mentioned above), which operated at higher pressures. Industry built upon the group’s work in this development–both the tools developed by the group (in particular, the REFPROP^4^ database described in section 3.5) and also the fundamental understanding of refrigerant mixture behavior.

#### 2.4.3. Other Alternatives.

Even as the HFCs were being commercialized in the 1990s and before GWP was an explicit concern, the search for alternative fluids continued, and a wide variety of chemical classes were considered. Kopko^45^ examined patterns in the properties of halogenated derivatives of dimethyl ether, cyclopropane, and propane, and suggested alternatives for R-11, R-12, R-113, and R-114. In the early 1990s, the Research Institute of Innovative Technology for the Earth (RITE) in Japan administered a major program involving government, academic, and industrial laboratories. They initially considered 500 compounds, with 70 being synthesized for further characterization; these included fluorinated alcohols and ethers, as well as compounds based on nitrogen and silicon. Three fluorinated ethers were the subject of detailed study.^46^ A somewhat analogous (although smaller) effort in the United States sponsored by the Electric Power Research Institute and carried out at Clemson University and the University of Tennessee synthesized a number of fluorinated ethers (see, for example, Beyerlein et al.,^47^ Kul et al.,^48^ Salvi-Narkhede et al.^49^). A range of iodine-containing compounds was investigated by Nimitz and Lankford.^50^

Bivens and Minor^51^ describe these efforts and others, including investigations of sulfur-based compounds and historical investigations of halogenated ethers, but concluded (in 1998) that “at this stage of the evaluations none appear to have a balance of refrigerant fluid requirements to challenge HFCs.” Interestingly, there was no mention of GWP in this article.

Also, in this period there was a resurgence of interest in the long-known natural fluids (e.g., ammonia, hydrocarbons, and carbon dioxide). In contrast to the compounds mentioned in the preceding two paragraphs, this interest has resulted in systems ranging from household refrigerators to industrial refrigeration systems based on the natural refrigerants.

### 2.5. Response to Global Warming.

As mentioned in the previous section, the contribution of refrigerants to global warming was known in the 1980s. Most of the HFCs have substantially lower values of GWP compared to the CFCs that they replaced. The urgent need at the time was to replace the ozone-depleting substances, and the GWP of the HFCs was felt to be “low enough.” (Note that the concept of “GWP” was not formally introduced until 1990.^52^ The GWP is a measure of the climate impact of a gas relative to that of carbon dioxide. It is calculated over some “time horizon,” and the value for a time horizon of 100 years is most commonly used and is referred to as GWP_100_.) For example, the GWP_100_ of the most common refrigerant in automotive systems was reduced by a factor of nearly eight (10200 to 1300) when R-12 was replaced with R-134a. Thus, the Montreal Protocol, which was originally targeted at stratospheric ozone protection, has already had a tremendously positive effect on climate, as reported by Velders et al.^53^ In that analysis, Velders et al.^53^ estimated that the Montreal Protocol has delayed climate change by 7−12 years.

Nevertheless, the use of the HFCs has continually increased since their introduction, and the “F-gas” regulations of the European Union were the first to regulate the HFCs. The first set of regulations^54^ and the associated Mobile Air Conditioning Directive^55^ mandated refrigerants with a maximum GWP_100_ of 150 in automotive systems. These were supplanted in 2014 by updated regulations,^56^ which mandate maximum values of GWP for refrigerants in most applications. Analogous regulations in the United States, known as SNAP^57^ (Significant New Alternatives Program) prohibit or allow specific refrigerants in various applications.

Velders et al.^58,59^ estimated that the HFCs would contribute 0.28 to 0.52 K to warming at the Earth’s surface by the end of the century in a “business as usual” scenario. Such an impact provided the impetus for a global agreement to reduce HFC emissions. This was negotiated within the framework of the Montreal Protocol, and the resulting 2016 Kigali Amendment to the Protocol^60^ called for an eventual 85% reduction in HFCs. It entered into force January 1, 2019. Velders estimated that Kigali would limit the climate impact of the HFCs to 0.06 K by the end of the century.^61^ The search was (and currently still is) on to find replacements for the replacements, and we are now in the “fourth generation” of refrigerants, defined by Calm^3^ as “global warming.” Kujak and Schultz^62^ provided an expanded set of environmental, safety, and sustainability objectives for any new refrigerant. These include considerations around product sustainability, such as long operational life, recyclable content, and minimized material use. It is also interesting to note that they cite “de minimis ODP” and “low flammability” rather than zero ODP and nonflammable as essential constraints.

As with the CFC phaseout of the 1980s and 1990s, the refrigerant manufacturers drew from their stock of known chemicals and offered possible replacements to the refrigeration industry. These were primarily hydrofluoroolefins (HFOs)–compounds with a carbon−carbon double bond, which greatly reduced their atmospheric lifetimes and, thus, GWP. The first round of the E.U. F-gas regulations focused on automotive air conditioning, and in response, SAE International (formerly known as the Society of Automotive Engineers) sponsored two successive “Cooperative Research Programs” to investigate refrigerants that would meet the F-gas target of GWP_100_ < 150. The initial interest in R-1234yf stemmed from these programs (see Brown^63^ for a summary). But, R-1234yf was known long before this. The synthesis of R-1234yf was first reported in the literature in 1946 by Henne and Waalkes^64^ for the purpose of studying interatomic distances; it was cited in a 1961 patent by Lo^65^ as a polymer precursor.

By 2008 the HFO refrigerants were frequently the topic of seminars at ASHRAE and other conferences. In particular, R-1234yf was being tested in a variety of equipment–not just the automotive systems for which it was originally developed. Again, the question arose: Are these the best fluids?

#### 2.5.1. NIST “Exploration of Thermodynamic Space”.

Beginning in 2012 McLinden, Kazakov, Domanski, and co-workers at NIST (NBS became NIST in 1988) undertook a major study to systematically search for suitable refrigerants. It had three major elements: (1) the identification of the fundamental thermodynamic characteristics of “ideal” refrigerants; (2) a search within a comprehensive chemical database to identify molecules having the defined characteristics; this included the estimation of properties based solely on chemical structure; and (3) simulation of the identified candidates in a representative vapor compression cycle. In many ways this represented a reprise of the 1987 study of McLinden and Didion,^42^ but with the advantage of much more sophisticated methods.

First of all, what are the thermodynamic characteristics of an “ideal” refrigerant? McLinden et al.^66^ and Domanski et al.^67^ approached this problem by defining fluids in terms of a small number of fundamental thermodynamic characteristics and then searching for optimal values of those parameters. Thus, they considered the full range of possible thermodynamic behavior, rather than scanning a finite number of known fluids (which would reveal no new fluids). They termed this approach the “exploration of thermodynamic space.”

The thermodynamic properties of a range of hypothetical fluids were modeled by the extended corresponding states (ECS) approach (summarized in section 3.2.2). The critical point parameters, *T*_c_ and *p*_c_, the heat capacity of the vapor (approximated by the ideal-gas heat capacity *C*_*p*_^0^), and the acentric factor, ω, which is related to the slope of the vapor pressure curve, were the primary thermodynamic parameters considered. Table 2 lists the ranges of these parameters considered. (Other parameters were considered, but they were found to be of minor importance.^67^)

The optimum thermodynamic parameters were found relative to objective functions of coefficient of performance (COP) (i.e., energy efficiency) and volumetric capacity (*Q*_vol_) for a cycle operating between an evaporation temperature of 10 °C and a condensation temperature of 40 °C. The hypothetical fluids were simulated in three cycles: (a) the simple (basic) vapor-compression cycle with four major components (compressor, condenser, expansion device, and evaporator), (b) a cycle with a liquid-line/suction-line heat exchanger, and (C) a two-stage economizer cycle. All three cycles were modeled in an ideal cycle assuming isentropic compression, no pressure drop in the heat exchangers, and saturated liquid and vapor exiting the condenser and evaporator, respectively. Sets of thermodynamic parameters within the ranges specified in Table 2 defined a series of hypothetical fluids. By varying these parameters according to an evolutionary algorithm, optimal values were determined; see McLinden et al.^66^ and Domanski et al.^67^ for details.

This exploration of thermodynamic space indicated a trade-off between efficiency and volumetric capacity. Refrigerants with a high critical temperature gave high efficiency, but low capacity; fluids with a relatively low *T*_c_ resulted in the converse. A critical pressure at the upper limit of the range resulted in both higher efficiency and increased capacity, while an acentric factor near the lower limit was optimal. The optimum value of the vapor heat capacity varied with the cycle; a relatively low value was best for the simple vapor-compression cycle, while a higher value was optimal for a cycle with internal heat exchange between the condenser outlet and compressor inlet.

Having defined a desired set of thermodynamic parameters, the next step was to identify fluids having those characteristics. The search relied on screening a comprehensive database of molecules by applying filters representing different refrigerant selection criteria. The search was carried out in the PubChem database–a listing with more than 60 million chemical structures.^68^ A first screening of this database is described by Kazakov et al.;^69^ we summarize here a second screening.^66^ All current refrigerants are small molecules, and the search was limited to molecules with 18 or fewer atoms and comprising only the elements C, N, O, S, H, F, Cl, or Br. The choice of elements follows the observation by Midgley^15^ that only a small portion of the periodic table would form compounds volatile enough to serve as refrigerants. These restrictions on elements and molecular size resulted in 184 000 molecules to be considered further.

Further screens for 320 K < *T*_c_ < 420 K and GWP_100_ < 1000 yielded 138 fluids. The PubChem database does not provide *T*_c_ and GWP_100_ for the vast majority of the compounds, so they were estimated using methods based solely on molecular structure, as described by Kazakov et al.^66,69–71^ The limits on critical temperature correspond to fluids usable in small AC systems, with an allowance for the uncertainty in the estimated values of *T*_c_. While refrigerants with values of GWP as low as possible are obviously desirable, fluids with GWP_100_ < 750 are, for example, permitted under E.U. regulations in AC systems with less than 3 kg of refrigerant.^56^ (The full list of 138 fluids is given in the Supporting Information of McLinden et al.,^72^ which also lists the estimated *T*_c_ and GWP_100_ for each fluid.)

The next screens were for chemical stability and toxicity. Compounds with generally unstable functional groups were dropped from further consideration. Attempts to automate the screening of toxicity were not successful, but, at this point, the number of compounds was sufficiently small to allow a “manual” examination of toxicity data.

The 138 candidates identified in the database screening were simulated in the simple (ideal) vapor-compression cycle; these used detailed equations of state (EOS) implemented in the NIST REFPROP database^73^ where available. However, for a majority of fluids the ECS model was used. This screening proceeded in two rounds. The first round of cycle simulations made use of the theoretical CYCLE_D model^74^ and provided a first estimate of volumetric capacity and COP.^75^ These simulations assumed an ideal cycle with 100% compressor efficiency and no pressure drops. Fluids with a low COP or volumetric capacity were dropped; this, combined with the screens for stability and toxicity, resulted in a list of 29 fluids. (The original list of McLinden et al.^72^ had 27 fluids; R-1132a was added because of revised toxicity data and CF_3_I was added because of commercial interest.)

The final list of 29 candidates is given in Table 3. This list is a subset of the 138 candidates having 320 K < *T*_c_ < 420 K and GWP_100_ < 1000, with the deletion of those that have low *Q*_vol_, low COP, or are unstable or toxic. The fluids are grouped by chemical class and, within classes, listed in order of increasing critical temperature; table adapted from ref.^72^ The list comprises four hydrocarbons and the closely related dimethyl ether; five fluorinated alkanes (i.e., HFCs); 10 fluorinated alkenes and an alkyne; two fluorinated oxygen-containing molecules; three fluorinated nitrogen or sulfur compounds; CF_3_I; and two inorganic molecules (ammonia or NH_3_ and carbon dioxide). The list includes a small number of novel molecules that have not been previously considered as refrigerants (at least publicly), but a majority of the fluids are well-known, including ammonia (R-717) and propane (R-290), or are the focus of current research in the refrigeration industry, that is, the fluorinated alkenes (HFOs).

**Figure 6.**
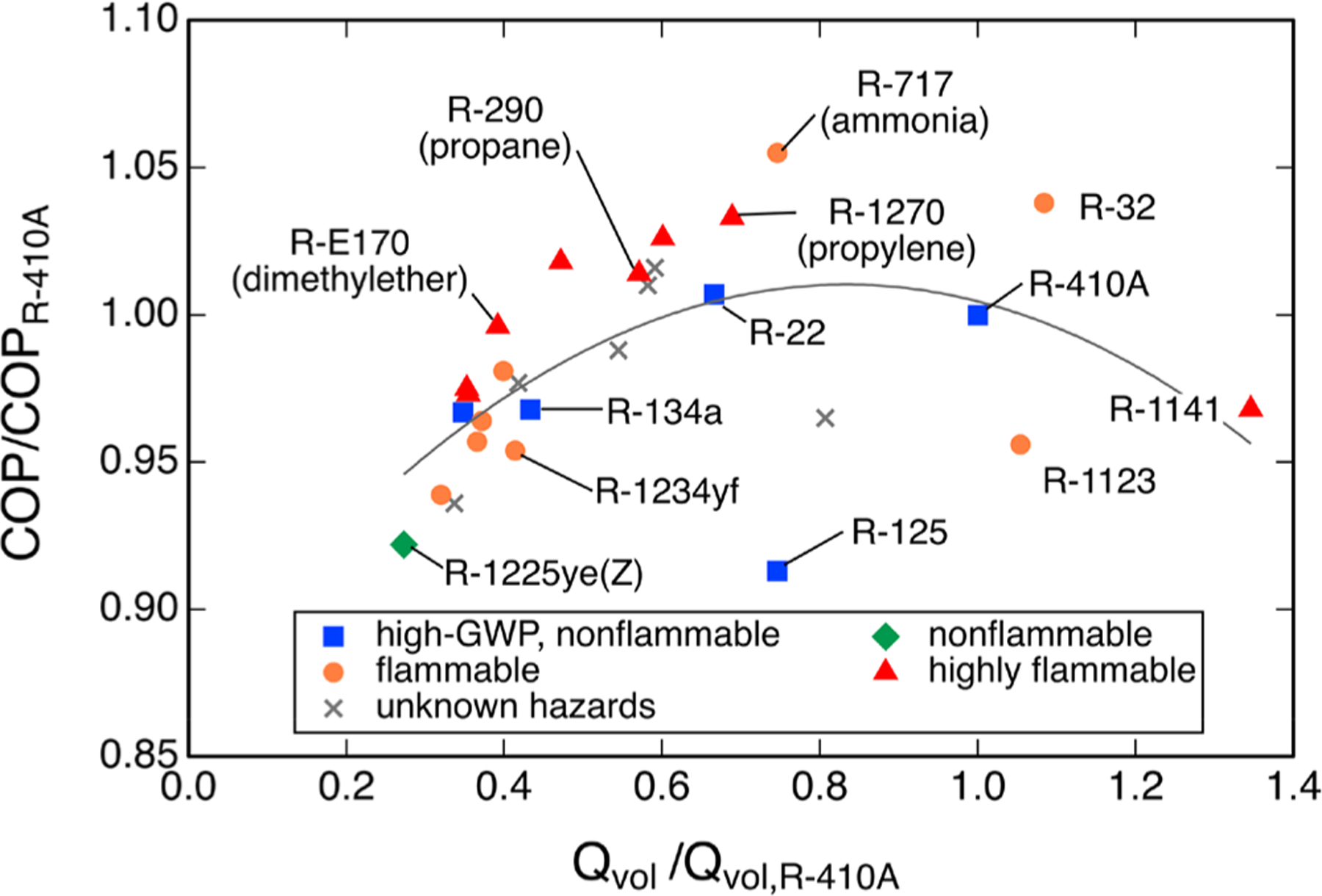
COP and *Q*_vol_ of selected low-GWP fluids relative to R-410A in the basic vapor-compression cycle including pressure drop and heat-transfer limitations; the curve indicates the general trend. Figure adapted from ref 72.

A second round of simulations made use of a more advanced “optimized” cycle model that provided a more realistic representation of an air conditioner employing typical forced-convection, air-to-refrigerant heat exchangers, which were optimized for a particular refrigerant.^76^ The COP and *Q*_vol_ of the candidate fluids, based on the optimized model, are presented in Figure 6. Unlike the COP versus *Q*_vol_ trade-off observed for the ideal analysis, the results of the optimized cycle simulations show a maximum in COP corresponding to *Q*_vol_ of approximately 60% to 110% that of R-410A. Although there is considerable scatter, a polynomial curve fitted to the fluids shown in Figure 6 indicates the general trend. Relative to fluids with low values of *Q*_vol_, the high-*Q*_vol_ fluids operate at higher pressures; consequently, pressure drops in the condenser and evaporator extract a smaller COP penalty, more than offsetting the inherently lower thermodynamic efficiency of operating closer to the critical point. This explains the current interest in finding relatively high-pressure refrigerants with properties similar to R-410A.

#### 2.5.2. Low-GWP Blends.

While the HFO refrigerants possess very low values of GWP, most of them come with the very significant trade-off of flammability. Many of the HFOs are in the “lower flammability” classification of “2L”. Nevertheless, as discussed in section 2.3, safety codes place (sometimes severe) restrictions on the use of flammable refrigerants. A further trade-off is that no nonflammable pure fluid can match the properties of R-410A.

The refrigeration industry has responded by proposing blends of the HFOs with nonflammable fluids to yield “class 1” blends with properties similar to R-410A but with reduced values of GWP. The blending agents are most often R-134a and R-125. As of January 2020, ASHRAE Standard 34 and its Addenda list 24 blends containing some combination of R-1234yf, R-1234ze(E), R-134a, and R-125. Many of these are summarized by Kujak and Schultz,^62,77,78^ but to suppress flammability relatively high concentrations of R-134a and/or R-125 are required, such that the 17 blends with a flammability classification of “1” have GWP_100_ values no lower than about 540, with 12 of the 17 having GWP_100_ > 1000. This is clearly an improvement over R-410A (GWP_100_ = 1924) and especially R-404A (which is used in commercial refrigeration systems and has a GWP_100_ = 3943). But, considering that the average tonnage-weighted GWP_100_ of refrigerants in HVAC use was 1768 (as of 2015) according to Booten et al.,^43^ future refrigerants must have an average GWP_100_ less than about 265 to meet the requirements of the Kigali Amendment to the Montreal Protocol, even ignoring future growth.

A second strategy for formulating a blend to replace R-410A is to accept a “2L” flammability classification in exchange for a pressure similar to R-410A and a much-reduced GWP_100_. There are currently 17 refrigerant blends in ASHRAE Standard 34 in this category, including 10 with R-32 as a major component.

There is clearly the need for an agent that would suppress flammability while having a very low GWP_100_. CF_3_I (also known as R-13I1 beginning with the 2019 version of ASHRAE Standard 34), with GWP_100_ < 1 is one such candidate. CF_3_I and other iodine-containing compounds were investigated in the 1990s during the search for CFC replacements (see Calm^6^ and Nimitz and Lankford^50^) and again in the early 2000s in the program sponsored by SAE International to identify replacements for R-134a in automotive air-conditioning systems.^63^ Interest waned in the iodine-containing compounds on both occasions because of stability and toxicity concerns, but there is now renewed interest in R-13I1 because of limited other options. The blend R-466A (consisting of R-32/125/13I1 with a composition of (49.0/11.5/39.5) mass percent) with a safety group classification of “A1” was added to Standard 34 in November 2019. The commercial viability of this blend may hinge on the ability of proprietary compounds added to stabilize the R-13I1.

## 3. EVOLVING PROPERTY MODELS AND THE REFPROP DATABASE

### 3.1. Early Approaches.

While equations of state (EOS) date back to the ideal-gas law, as first stated by Clapeyron^79^ in 1834, and the van der Waals^80^ EOS (1873), early calculations of refrigeration cycles inevitably used tables or charts of thermodynamic properties. The ammonia tables prepared in 1923 by the Bureau of Standards^81^ (now NIST) are a prime example. Faced with inconsistent ammonia tables from a number of sources, the American Society of Refrigeration Engineers (now ASHRAE) lobbied the U.S. Congress to fund a program at the Bureau of Standards to prepare a definitive set of properties.^82^ Work commenced in 1915 with federal funding and continued with industry funding. The Bureau carried out an extensive program of measurements on the vapor pressure, density of liquid and vapor, latent heat of vaporization, and heat capacity of liquid and vapor that were state of the art for the day (and remain among the best data for ammonia to this day). The EOS of the time could not represent the data within their experimental uncertainties; thus, separate empirical equations for the specific volume, enthalpy, and entropy of the vapor phase were fitted to the measured data. Additional equations for the saturated liquid and compressed liquid “under moderate pressure” were also developed. These equations were then used to compute (at a time when a “computer” was a person operating a mechanical calculator limited to basic arithmetic) a set of tables that covered 30 pages as well as a fold-out “Mollier diagram.” These remained the accepted properties for ammonia until they were supplanted by an equation-of-state approach developed at the National Bureau of Standards in 1978.^83^

The reign of tables and diagrams continued well into the era of the CFCs and HCFCs. These were often prepared by the refrigerant manufacturers. A well-known example was the “Technical Data Bulletins” of the DuPont Company. For example, the bulletin on R-22^84^ provided a brief summary of new experimental measurements carried out at university laboratories. The vapor-phase properties were calculated by an equation of state, which was an empirical extension of the van der Waals EOS. The EOS was supplemented by separate equations for the vapor pressure, saturated-liquid density, and vapor heat capacity. The bulk of the bulletin, however, was given over to 42 pages of detailed tables. That particular bulletin was first published in 1964 but was reprinted well into the 1980s. Books with extensive tables continued to be published into the 1990s; examples include Baehr and Tillner-Roth^85^ and Tillner-Roth et al.^86^

### 3.2. Equations of State.

Equations of state date back to the ideal gas law,

(1) P=RT/V

where *P*, *V*, and *T* are pressure, molar volume, and temperature, respectively, and *R* is the gas constant. To represent both gas and liquid phases, “cubic” equations of state were developed (they can be expressed as a function that is cubic in volume). The earliest one was by van der Waals^80^ in 1873:

(2) $$P=RT/\left(V-b\right)-a/V^{2}$$

where parameter *b* is an excluded volume, and *a* is a parameter to account for attractive forces between molecules. These act as corrections to the ideal gas law, which assumes there are no intermolecular forces and that the molecules have negligible volume. The van der Waals equation has behavior that is qualitatively correct but cannot quantitatively represent many saturation and single-phase properties for fluids, including even simple ones such as argon. It lacks the ability to predict liquid-phase and saturation densities with reasonable accuracy, and the representation of properties for polar fluids is even worse.^87^ A major improvement came in 1949 with the Redlich−Kwong^88^ (RK) equation of state. Similar to the van der Waals EOS, the RK EOS can be expressed as cubic in volume, and it has two parameters that are constants that can be related to the critical parameters. Both EOS are the sum of an attractive contribution and a repulsive contribution; however, the Redlich−Kwong EOS introduced temperature dependence into the attractive term, and has the form

(3) $$P=RT/\left(V-b\right)-a/\left[T^{0.5}V\left(V+b\right)\right]$$

Later in the 1970s two additional cubic equations of state, the Peng−Robinson^89^ and the Soave−Redlich−Kwong^90^ equations became widely used by engineers, especially in the oil and gas industry. These equations added more flexible temperature dependence to the attractive parameter *a*. There are now a very large number of cubic equations of state; they continue to be developed and used, including a recent application to HFCs and HFOs.^91^ A review of the cubic EOS is provided by Lopez-Echeverry et al.^92^

The refrigeration industry, however, often used the Martin-Hou^93^ equation of state, which retains the van der Waals repulsive term but introduces a more complex attractive term:

(4) $$P=RT/\left(V-b\right)+\sum_{i=2}^{5}\frac{A_{i}+B_{i}T+C_{i}\text{exp}\left(-KT/T_{c}\right)}{{\left(V-b\right)}^{i}}$$

where *K*, *A*_*i*_, *B*_*i*_, and *C*_*i*_ are fitted parameters. Unlike the cubic EOS, it is not valid in the liquid region and requires ancillary equations for vapor pressure and saturated liquid density. An extensive discussion of this EOS and its application to early refrigerants is provided by Downing.^94^

#### 3.2.1. Carnahan−Starling-DeSantis EOS.

In the mid-1980s researchers at NBS involved with alternative refrigerants began working with the Carnahan−Starling-DeSantis^95^ (CSD) equation of state, expressed as

(5) $$PV/RT=\left(1+y+y^{2}-y^{3}\right)/{\left(1-y\right)}^{3}\;\;-a/\left[RT\left(V+b\right)\right],\;y=b/4V$$

The CSD model was selected for several reasons. At that time, there often were very few reliable data for thermodynamic properties of new refrigerants. The CSD equation has a theoretical basis and needs few data to obtain parameters. It is simple yet can represent both the vapor and liquid phases. It not only correlates whatever limited data exist, but because it has a sound theoretical basis, it provides thermodynamic consistency across properties, realistic limiting behavior, and the ability to predict properties, especially for mixtures for which the data are even more limited than for pure fluids. Morrison and McLinden^96^ modified the CSD EOS slightly by introducing temperature-dependent functions for the parameters *a* and *b*:

(6) $$a=a_{0}\exp\left(a_{1}T+a_{2}T^{2}\right)\;\text{and}\;b=b_{0}+b_{1}T+b_{2}T^{2}$$

This equation has a total of six parameters that typically are determined from fitting saturated liquid and vapor volumes and the vapor pressure.

#### 3.2.2. Extended Corresponding States Model.

As research on alternative refrigerants intensified in the 1980s, there often was a lack of experimental property data. A modeling approach that can be used to obtain properties when data are scarce or unavailable is the extended corresponding states (ECS) model, originally proposed by Leland and co-workers^97,98^ in the late 1960s and early 1970s. It had been used at NIST to represent the properties of hydrocarbon fluids and their mixtures,^99,100^ and later was modified slightly to apply to refrigerants.^101^ The basic idea of simple corresponding states^102,103^ is that if two fluids are conformal (i.e., they obey the same intermolecular force law expressed in reduced variables) the reduced residual Helmholtz free energy $\alpha^{\text{r}}=\left[A\left(\rho ,T\right)-A^{\text{id}}\left(\rho ,T\right)\right]/RT$ and the residual compressibility factor $z^{\text{r}}=\left(PV/RT-1\right)$ may be written

(7) $$\alpha_{j}^{\text{r}}\left(T_{j},\rho_{j}\right)=\alpha_{0}^{\text{r}}\left(T_{0},\rho_{0}\right)\;\text{and}\;z_{j}^{\text{r}}\left(T_{j},\rho_{j}\right)=z_{0}^{\text{r}}\left(T_{0},\rho_{0}\right)$$

where *A* is the molar Helmholtz energy and *ρ* is the molar density; the subscript *j* denotes the fluid of interest, and 0 refers to a reference fluid. “Residual” refers to a property minus that property in the limit of zero density. Scaling factors *f*_*j*_ and *h*_*j*_ relate the properties of the fluid *j* to the reference fluid 0,

(8) $$T_{0}=T_{j}/f_{j}\;\text{and}\;\rho_{0}=\rho_{j}h_{j}$$

In other words, with the ECS approach one can use the properties of a well-known reference fluid to compute the properties of an unknown fluid with only a few additional parameters. For simple fluids that are spherically symmetric, the scaling factors are ratios of the critical parameters of the fluids,

(9) $$f_{j}=T_{c,j}/T_{c,0}\;\text{and}\;h_{j}=\rho_{c,0}/\rho_{c,j}$$

The simple corresponding states, as expressed above, are valid only for an extremely limited number of molecules that are spherically symmetric, such as argon. Most refrigerants are generally nonspherical and often polar. To address this problem, Leland and Chappelear^97^ introduced the concept of “extended” corresponding states, where “shape factors” *θ*_*j*_ and *ϕ*_*j*_ are introduced:

(10) $$f_{j}=\left(T_{\text{c},j}/T_{\text{c},0}\right)\theta_{j}\left(T,\rho \right)\;\text{and}\;h_{j}=\left(\rho_{\text{c},0}/\rho_{\text{c},j}\right)\varphi_{j}\left(T,\rho \right)$$

The shape factors are functions of both temperature and density (although one can sometimes ignore the density dependence as was done in ref 101) and can be obtained in different ways depending on the amount of experimental data available and the assumptions made.^101,104^

For refrigerants, R-134a (1,1,1,2-tetrafluoroethane) was selected as a reference fluid because there was a large amount of experimental data and good models for both the thermodynamic and transport properties. In addition, when in predictive mode, it works best if the fluid of interest is as chemically similar as possible to the reference fluid; R-134a was a good choice for emerging methane or ethane-based HCFCs and HFCs.

#### 3.2.3. Modified Bennedict−Webb−Rubin EOS.

Some of the third-generation refrigerants, such as R-134a, were more polar than the second generation CFCs and HCFCs such as R-12 and R-22 that they were designed to replace. This presented difficulties with the use of the CSD EOS, and an alternative EOS, the modified Benedict−Webb−Rubin (MBWR) EOS, began to be used to fit the data more accurately. It was significantly more complex than the simple CSD EOS, expressing pressure as an explicit function of temperature and molar density with the form:

(11) $$P=\sum_{n=1}^{9}\alpha_{n}\rho^{n}+\exp\left[{\left(\rho /\rho_{\text{c}}\right)}^{2}\right]\sum_{n=10}^{15}\alpha_{n}\rho^{2n-17}$$

where the *α*_*i*_ are simple functions of temperature, resulting in a total of 32 parameters. The MBWR EOS (or any pressure-explicit EOS) must be combined with an expression for the molar heat capacity of the ideal gas *C*_*p*_^0^ to compute energy functions such as enthalpy and entropy. With an expression for *C*_*p*_^0^, all thermodynamic properties may then be computed, as described in ref.^105^

#### 3.2.4. Fundamental EOS Based on Helmholtz Energy.

The equations of state discussed so far (with the exception of ECS, which technically is a model, not an EOS) are written in the form *P* = *f*(*T*, *ρ*), as it is quite natural to use pressure, density, and temperature because they are quantities that are easily measured, and using this form has a tradition going back to the ideal gas law. When an equation of state is expressed in the form *P* = *f*(*T*, *ρ*), and one has an expression for *C*_*p*_^0^(*T*), all thermodynamic properties can be computed, but integration is required to obtain caloric properties. Obtaining analytical expressions for such EOS is not problematic, but it does impose restrictions on the types of terms that can be used. If one instead uses a formulation in terms of the residual Helmholtz energy, *A*^r^, there is much more flexibility in the types of terms that can be used, because all thermodynamic properties can be obtained from the Helmholtz energy by taking derivatives rather than integrals. In the mid 1980s researchers, including the groups of Wagner^106^ and of Jacobsen,^107,108^ began using optimization algorithms with multiproperty fitting to determine the terms in an equation of state, and the use of the Helmholtz form gave them flexibility to develop new forms that were not possible with pressure-explicit formulations. In the 1990s these Helmholtz formulations, also called “fundamental” EOS, began to be made for refrigerants, including R-11,^109,110^ R-12,^110^ R-22,^111^ R-32,^112^ R-113,^110^ R-124,^113^ R-143a,^114^ and R-134a.^115^

This type of EOS expresses the reduced molar Helmholtz free energy *α* in terms of a reduced temperature and reduced density and often takes the form:

(12) $$\alpha \left(\delta ,\tau \right)=\frac{A}{RT}=\alpha^{\text{id}}+\alpha^{\text{r}}=\alpha^{\text{id}}+\sum N_{k}\delta^{d_{k}}\tau^{t_{k}}+\;\;\sum N_{k}\delta^{d_{k}}\tau^{t_{k}}\exp\left(-\delta^{l_{k}}\right)$$

where the *α*^id^ is the ideal gas (zero-density) contribution, and *α*^r^ is the residual, or real fluid contribution. More recent forms include additional terms to allow for better representation of the critical region,^116^

(13) $$\alpha^{\text{r}}\left(\delta ,\tau \right)=\sum N_{k}\delta^{d_{k}}\tau^{t_{k}}+\sum N_{k}\delta^{d_{k}}\tau^{t_{k}}\exp\left(-\delta^{l_{k}}\right)\;\;\;\;\;+\sum N_{k}\delta^{d_{k}}\tau^{t_{k}}\exp\left(-\delta^{l_{k}}\right)\exp\left(-\tau^{m_{k}}\right)$$

The temperature and density are expressed in reduced variables *τ* = T*/*T* and *δ* = *ρ*/ρ* where T* and ρ* are reducing parameters that often are the critical parameters. The *N*_*k*_ are coefficients obtained by fitting experimental data, and the exponents *d*_*k*_, *t*_*k*_, *l*_*k*_, and *m*_*k*_ are also determined by regression. Each summation typically contains 4 to 20 terms, and the index *k* points to each individual term. An advantage of this form is that thermodynamic properties can be expressed in terms of derivatives, rather than integrals, for example

(14) $$\frac{P}{\rho RT}=1+\delta {\left(\frac{\partial \alpha^{r}}{\partial \delta }\right)}_{\tau }\;\text{and}\;c_{v}/R=-\tau^{2}{\left(\frac{\partial^{2}\alpha }{\partial \tau^{2}}\right)}_{\delta }$$

Expressions for additional properties can be found in the Appendix of ref 116.

Traditionally, the fitting started with a large bank of terms, and unnecessary terms were eliminated until an optimal form was found. With faster computers a fully nonlinear least-squares fit could be implemented, which allowed terms of an arbitrary form. Further advances use multiple constraints to reduce the total number of terms, control the extrapolation behavior, improve the behavior in the two-phase region, and ensure physically realistic behavior in all regions of the fluid. For a more detailed discussion of multiparameter Helmholtz equations of state the reader is referred to Lemmon and Jacobsen^116^ and to the book by Span.^117^

#### 3.2.5. International Energy Agency Annex 18 and “Refrigerant Olympics”.

Just as the refrigeration industry faced inconsistent ammonia tables in the early 1900s (as described in section 3.1) multiple formulations for R-134a, R-123, and other “new” refrigerants were appearing by the late 1980s. There was little coordination of effort–many groups were measuring the properties of a few leading alternative fluids, while there was a dearth of data on others. A small group of researchers approached the International Energy Agency (IEA) to sponsor a working group (known as an “Annex” in IEA lingo) to coordinate efforts, provide a forum for the exchange of information, and ultimately to endorse property formulations as international standards. This group, known as “Annex 18–Thermophysical Properties of the Environmentally Acceptable Refrigerants” operated from 1990 to 1999; its activities are summarized by McLinden and Watanabe.^118^ Participants from eight member countries (with observers from three other countries) met regularly in North America, Europe, and Japan; they included many of the leading properties research groups from around the world.

A major activity of the Annex was a series of EOS evaluations. Modeling groups were invited to submit EOS, which were then evaluated and compared to other “entrants” in what came to be called the “Refrigerant Olympics.” R-134a and R-123 were the first to be considered, followed by R-32, R-125, and R-143a. The evaluations were carried out by experienced EOS groups who compared the equations to a consistent experimental data set using a consistent set of statistical measures; this process is described by Penoncello et al.^119^ The winners were endorsed as international standards with the EOS documented in the *Journal of Physical and Chemical Reference Data*.^105,112,115,120,121^ The Annex did not have any formal authority to declare international standards but operated on the belief that consensus could be quickly reached by demonstrating high accuracy in a transparent process. The evaluation process established a high standard, which raised the bar for refrigerant EOS; it also established the Helmholtz-energy based equations as the most accurate EOS (four of the five EOS endorsed as standards were Helmholtz based) at a time when they were just coming into widespread use.

### 3.3. Extension to Mixtures.

All of the equations of state and models discussed in section 3.2 can be extended to mixtures. There are several approaches to modeling a mixture. One is to treat the mixture at a fixed composition as a pure fluid, such as has been done with R-404A, R-410A, R-507A, and R-407C.^122^ This approach has the disadvantage that accounting for variations in properties as a function of composition is not possible.

A second approach is to use mixing rules applied to the various parameters in the pure fluid equation of state. For example, for the Redlich−Kwong EOS^88^ there is a mixing rule for the *a* and *b* parameters, with combining rules for the cross term *a*_*ij*_

(15) $$a_{\text{mix}}=\sum_{i=1}^{n}\sum_{j=1}^{n}x_{i}x_{j}a_{ij}\;\text{and}\;b_{\text{mix}}=\sum_{i=1}^{n}x_{i}b_{i}$$

(16) $$a_{ij}=\sqrt{a_{i}a_{j}}$$

where the *x* are compositions in mole fraction, and the summation is over the *n* components in the mixture. Other cubic equations of state use similar expressions; some introduce a quadratic mixing rule and combining rules for *b* as well, and may also introduce binary interaction parameters that are found by fitting experimental data.

A third way to represent mixture properties using an equation of state approach is by applying mixing rules to a particular property. Lemmon^123^ and Tillner-Roth^124^ both developed mixture models based on finding the Helmholtz energy of the mixture,

(17) $$\alpha_{\text{mix}}=\sum_{j=1}^{n}\left[x_{j}\left(\alpha_{j}^{\text{id}}+\alpha_{j}^{\text{r}}\right)+x_{j}\ln x_{j}\right]\;\;\;+\sum_{p=1}^{n-1}\sum_{q=p+1}^{n}x_{p}x_{q}F_{pq}\alpha_{pq}^{\text{excess}}$$

where the first summation is the contribution from the EOS of each of the constituent pure fluids, the *x* ln *x* term accounts for the entropy of mixing, and the second summation represents the departure from ideal mixing. The *F*_*pq*_ terms are generalizing parameters that relate the behavior of one binary pair with that of another, it multiplies the $\alpha_{pq}^{\text{excess}}$ terms which are empirical functions that are fit to binary mixture data. Similar to corresponding states discussed in section 3.2.2, the *α*_*j*_ and $\alpha_{pq}^{\text{excess}}$ terms are not evaluated at the temperature and density of the mixture, *T*_mix_ and *ρ*_mix_, but rather at a scaled or reduced temperature and density *τ* = *T*^red^*/T*_mix_ and *δ* = *ρ*_mix_*/ρ*^red^. Mixing rules are used to determine the reducing values *T*^red^ and *ρ*^red^, for example, one set that was used for mixtures containing R-32, R-125, R-134a, R-143a, and R-152a is^125^

(18) $$\rho_{\text{red}}={\left[\sum_{i=1}^{n}\frac{x_{i}}{\rho_{c_{i}}}+\sum_{i=1}^{n-1}\sum_{j=i+1}^{n}x_{i}x_{j}\xi_{ij}\right]}^{-1}\text{and}\;\;T_{\text{red}}=\sum_{i=1}^{n}x_{i}T_{c,i}+\sum_{i=1}^{n-1}\sum_{j=i+1}^{n}x_{i}x_{j}\xi_{ij}$$

The parameters ζ_*ij*_ and ξ_*ij*_ are used to define the shapes of the reducing temperature and density curves. These reducing parameters are not the same as the critical parameters of the mixture and may be found by fitting experimental data or from a predictive model.^126^ There are other forms of mixing rules for Helmholtz-based mixture models, for example see refs 127 and 117.

### 3.4. Transport Property Models.

There are a variety of methods for the correlation, prediction, and estimation of transport properties of fluids, see for example, Millat et al.^87^ and Poling et al.^128^ There is no theory that can predict the transport properties of fluids over the entire range of temperature and pressure with an accuracy that is useful for engineering or scientific purposes. Thus, one must rely on empirical correlations or semiempirical models. We limit our discussion here to a model that has been successfully applied to refrigerants and their mixtures and is capable of representing the entire fluid surface.

The amount and quality of data for viscosity and thermal conductivity generally are much less than for thermodynamic properties. Given that fact, it is useful to have a model that can be used when data are sparse, or even nonexistent. It also is desirable to have a model that can represent properties across the entire fluid surface including gas, liquid, and supercritical states rather than separate correlations for saturated liquid, vapor, etc., and also can be applied to mixtures. One such model is the extended corresponding states model, which was discussed with respect to thermodynamic properties in section 3.2.2; it may be applied to transport properties as well.

In extended corresponding states the viscosity *η* of a pure fluid is represented as a sum of a dilute-gas value η*(*T*) and a residual contribution Δ*η*(*ρ,T*). Only the residual contribution is treated via corresponding states:

(19) $$\eta \left(T,\rho \right)=\eta *\left(T\right)+\Delta \eta \left(T,\rho \right)\;\;\;\;\;\;=\eta *\left(T\right)+\Delta \eta_{0}\left(T_{0},\rho_{0}\right)F_{n}\left(T,\rho \right)$$

The function *F*_*η*_ in eq 19 is found using the expression

(20) $$F_{\eta }\left(T_{j},\rho_{j}\right)=f_{j}^{1/2}h_{j}^{-2/3}{\left(M_{j}/M_{0}\right)}^{1/2}$$

where *M*_*j*_ is the molar mass of the fluid and *M*_0_ is the molar mass of the reference fluid. To improve the representation of the viscosity, an empirical correction factor may be introduced into eq 20 if experimental viscosity data are available.^129^ The procedure for the thermal conductivity is very similar and the reader is referred to Huber et al.^130^ for details. Thermal conductivity is more complicated than viscosity, however, because of the presence of an enhancement in the critical region that can be significant and should not be neglected.^131,132^

The application of this model to mixtures adds an additional step–one must first use mixing and combining rules to generate a hypothetical pure fluid to represent the mixture, and then the hypothetical pure fluid is mapped onto the reference fluid as described above. Details on this process can be found in numerous references.^101,130,133–135^ This model is often called a “one-fluid” model and works best for systems in which the components are generally similar in size and polarity. Unfortunately, this approach is not applicable at all to mixtures of very dissimilar fluids such as ammonia/water and alcohols/water. In addition, it has difficulties with refrigerant systems with components of widely differing polarities or sizes; however, when data are available, fitting binary interaction parameters can improve the results. Research on alternative models that can handle these systems is ongoing.

### 3.5. The NIST REFPROP Database.

Carrying out refrigeration cycle calculations by interpolating properties from tables worked well enough when designs were largely empirical and when only a single (pure-fluid) refrigerant was considered. But, with more sophisticated numerical modeling of refrigeration cycles, or when multiple fluids were under consideration to replace a CFC or, especially, refrigerant blends were being compared, a better tool was needed.

The CSD equation of state (discussed in section 3.2.1) was implemented as a set of Fortran subroutines as part of the NBS research program on refrigerant mixtures (section 2.4.2). These are described by Morrison and McLinden^96^ for 11 (mostly CFC) refrigerants, namely R-11, R-12, R-13, R-13B1, R-14, R-22, R-23, R-113, R-114, R-142b, and R-152a and their binary mixtures. These routines were distributed on magnetic tape (predating floppy disks) and were also listed in the printed document that was mailed out (no pdf’s in the 1980s).

With the increasing interest in the properties of “new” refrigerants and their mixtures in response to the need to find replacements for the CFCs, NBS/NIST decided to formalize the CSD subroutines into a “Standard Reference Database” called REFPROP (for REFrigerant PROPerties). The first version, released in December 1989 contained the original 11 refrigerants plus R-123, R-124, R-134, and R-134a. It was distributed on 5^1^/_4_ inch floppy disks for a DOS-based system, the state-of-the-art at that time. The disk contained the Fortran subroutines as well as a DOS-based user interface, which was extremely crude by modern standards.

**Figure 7.**
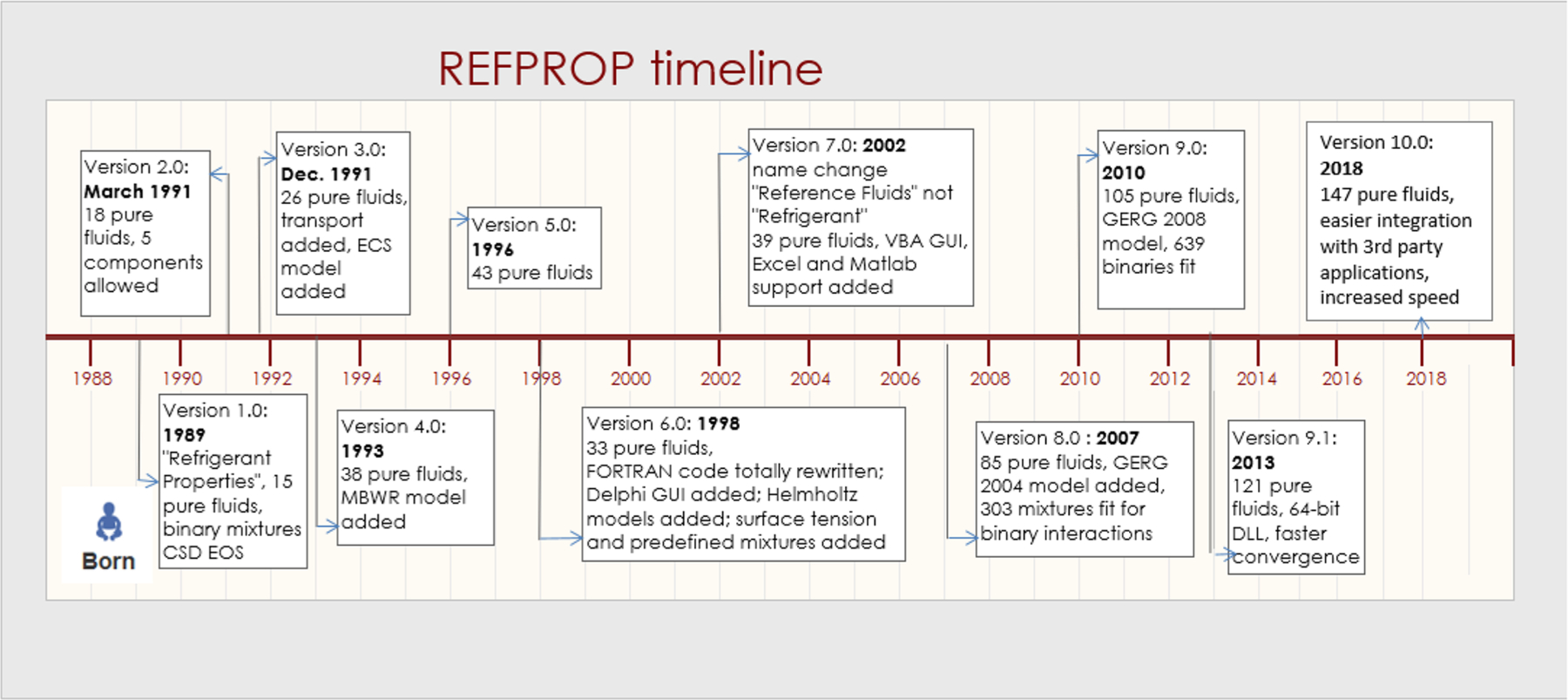
Timeline for REFPROP.^4^

Following its initial release in 1989, the REFPROP program underwent changes to incorporate developments in computer technology, alternative refrigerant research, and improvements in equations of state. Figure 7 shows a timeline. The second version, released in 1991, increased the number of pure fluids from 15 to 18. It also was no longer restricted to binary mixtures and allowed mixtures of up to five components. In 1991, version 3.0 added viscosity and thermal conductivity incorporating an ECS model as described in section 3.4.1. Some of the third-generation refrigerants, such as R-134a, were more polar than the second-generation CFCs and HCFCs, such as R-22, that they were designed to replace. This presented difficulties with the use of the CSD EOS, and an alternative EOS, the modified Benedict−Webb−Rubin (MBWR) EOS, began to be used to fit the data more accurately. When few data were available, the extended corresponding states model^101^ was used. REFPROP version 4, released in 1993, incorporated the new MBWR EOS, including formulations for R-134a^136^ and R-123.^105^

Version 5.0, released in 1996, added additional pure fluids to REFPROP. By this time, the DOS-text-only based interfaces had become archaic, and work was begun to replace it with a much more capable and user-friendly graphical user interface (GUI). This led to version 6.0 of REFPROP in 1998 with a GUI, written in the language Delphi. Also, at this time there were improvements to the method of finding the shape factors in the ECS transport model, as described in refs 133 and 134. Surface tension was also added. In addition, predefined refrigerant mixtures, such as R-404A and R-410A, were also added as industry increasingly looked to mixtures over individual pure fluids.

In 2002, with version 7.0, the name REFPROP was revised to stand for REference Fluid PROPerties, to reflect a change in scope, which widened the type of fluids covered. (By this time, much of the work on the HFC refrigerants had been completed, and the properties group at NIST turned their attention to other fluids.) Components of natural gas were added, as well as nitrogen, oxygen, argon, and normal and parahydrogen. Standards from the International Association for the Properties of Water and Steam (IAPWS) were incorporated for the properties of water. REFPROP now could essentially replace two earlier NIST computer programs that were aimed at the cryogens and natural gas communities (MIPROPS and DDMIX). Version 7.0 incorporated a GUI in Visual Basic and also added support for Excel and Matlab. Developments in equations of state were happening rapidly and were included in REFPROP. Instead of representing an equation of state in the familiar terms of pressure as a function of temperature and density, the Helmholtz energy formulations for EOS (as discussed in section 3.2.4) came into use.

Version 8.0, released in 2007, increased the number of pure fluids to 85. The new fluids added included primarily hydrocarbons such as heavier straight chain alkanes from *n*-pentane through *n-*decane, *n*-dodecane, alcohols (methanol and ethanol), aromatics (benzene, toluene), branched alkanes (isohexane, isopentane, neopentane), alkenes (1-butene, *trans*-2-butene), some cyclic alkanes (cyclopentane, cyclohexane), and an ether (dimethyl ether). With this slate of fluids, natural gas mixtures could be modeled and version 8 introduced a new model for natural gas fluids, the GERG-2004 (European Gas Research Group) model of Kunz et al.^127^ This model is based on an excess Helmholtz energy approach that uses pure fluid equations of state and a mixture model that describes the excess contribution. Binary interaction parameters are used to improve mixture calculations, and version 8.0 included interaction parameters for 303 mixtures that resulted from fitting experimental data.

In response to industry needs, additional fluids were added to REFPROP, version 9.0, in 2010. These included new low-GWP alternative refrigerants, the HFOs R-1234yf and R-1234ze(E). Two new classes of fluids unrelated to refrigerants were introduced in v9 as well: siloxanes and fatty acid methyl ethers (FAMES), which are components of soy-derived biodiesel. Version 9.0 included the updated natural gas standard, GERG-2008^137^ as well as additional fluids bringing the total number of pure fluids to 105 with additional binary interaction parameters. Three years later, version 9.1 saw the addition of more low-GWP alternatives including a fluorinated ketone (Novec-649), several fluorinated ethers (R-E143a, R-E245cb2, R-E245fa2, R-E347mcc), and a hydrochlorofluoroolefin (HCFO), R-1233zd(E).

The current version is version 10, which was released in 2018. New low-GWP refrigerants R-1123, R-1224yd(Z), R-1234ze(Z), R-1243zf, and R-1336mzz(Z) were added. All the pure-fluid refrigerants currently in REFPROP and the sources of property formulations are listed in the Supporting Information. New mixing parameters based on fitting data for binary mixtures with mixtures containing R-32, R-125, R-134a, R-1234yf, and R-1234ze(E) were included to replace a predictive model^126^ that was used in earlier versions. New models for mixtures such as ethylene glycol/water and ammonia/water were added, along with additional binary interaction parameters, improvements in speed, and easier interaction with third-party programs (such as Excel and MatLab).

REFPROP and all of the models described above rely, of course, on experimental data. A review of the extensive literature data on the refrigerants is beyond the scope of this paper, but we acknowledge the many contributions of institutions worldwide that have made significant contributions to the measurement and modeling of the thermophysical properties of alternative refrigerants. These include Ruhr-Universität Bochum, Universität Stuttgart, Friedrich-Alexander-Universität Erlangen-Nürnberg, Universität Rostock, Universität Bremen, and the Leibniz Universität Hannover (Germany); Xi’an Jiaotong University and Chinese Academy of Science (China); University of Idaho and Catholic University of America (USA); UniversitéParis (France); University of Padua, Italian National Research Council (CNR) (Italy); Imperial College London (UK); Aristotle University of Thessaloniki (Greece); IUPAC, Universidad de Valladolid and Universidad de Extremadura (Spain); University of Lisbon and Instituto Superior Tećnico (Portugal); Keio University, Tohuko University, National Institute of Advanced Industrial Science and Technology (AIST), Saga University, Kyushu University and Kyushu Sangyo University (Japan); Korea Institute of Science and Technology, Sogang University, and Seoul National University (Korea); and University of Western Australia (Australia).

The REFPROP program has now been in existence for over 30 years and has become the *de facto* refrigeration industry standard. It is under continual development to respond to industry needs as new refrigerants and their mixtures are identified.

## 4. DISCUSSION, CONCLUSIONS, AND OUTLOOK

Since the earliest days of mechanical refrigeration, the refrigerants in use have continually evolved in response to evolving constraints driven by changing types of equipment and changing demands on safety and environmental characteristics. Early systems employed a range of flammable and/or toxic fluids; they worked, but their hazards hindered the widespread adoption of refrigeration. In the late 1920s, Midgley arrived at the surprising realization that adding fluorine to a molecule could yield a nonflammable and low-toxicity refrigerant, thus launching the era of the CFCs and forever resetting expectations for the safety of refrigerants. By the 1980s the CFCs were clearly implicated in the destruction of stratospheric ozone, adding an environmental constraint. In response, the chemical industry identified the HFCs from their trove of molecules as replacements, and this generation of refrigerants is allowing the Antarctic ozone hole to heal, while maintaining safety. But the HFCs are potent greenhouse gases, and this additional environmental constraint presents the refrigeration industry with the difficult task of balancing trade-offs among GWP, ODP, flammability, stability, energy efficiency, and system complexity. An “exploration of thermodynamic space” revealed that there are no more silver-bullet molecules that can simultaneously satisfy all these constraints.

In some cases, this evolution has been revolutionary, as in the case of Midgley’s development of the CFCs. The evaluation of alternatives has progressed from searching among known compounds to the ability to evaluate virtually any molecule that could be imagined. But in other ways, “there is nothing new” in the sense that the fundamental thermodynamic characteristics that make a good refrigerant have not changed since ammonia was recognized as an excellent refrigerant in the 19th Century. Furthermore, seemingly “new” refrigerants were often identified in the chemical literature decades before their use as refrigerants. We also are seeing instances of refrigerants “revolving” back (i.e., circling back) to earlier choices (e.g., ammonia, CO_2_, hydrocarbons).

A similar evolution/revolution in property models parallels the development of the refrigerants themselves. The refrigeration industry has progressed from interpolating properties in tables and diagrams to calling wide-ranging, high-accuracy property models at any arbitrary temperature, pressure, and mixture composition. The NIST REFPROP database has evolved along with the refrigerants in contemporaneous use. In some cases, it led the way, providing, for example, a tool to evaluate refrigerant blends at a time when blends were unfamiliar to most in the industry. At other times, NIST scrambled to keep up with developments, such as when a new refrigerant was announced and there was a sudden demand to have its properties “yesterday.”

It is almost certain that refrigerants will continue to evolve in the future, and it will be ever-changing constraints that will drive this evolution. The molecules from which to choose are almost certainly known today. The constraints in the future could be relaxed (e.g., to allow more flammable fluids) or, perhaps, to allow a very small ODP in return for a much-reduced GWP; or they could become more stringent, drawing a wider “system boundary” on the environmental consequences of refrigerants to also include the inputs to their manufacture and/or the impact of their breakdown products in the atmosphere.

## Supplementary Material

Supp1

## Figures and Tables

**Table 1.** Refrigerant Criteria, as Given by McLinden and Didion^a^

Refrigerant Criteria
** ● Chemical**
○ Stable and inert
** ● Health, Safety and Environment**
○ Nontoxic
○ Nonflammable
○ Does not degrade the atmosphere
** ● Thermal (Thermodynamic and Transport)**
○ Critical point and boiling point temperatures appropriate for the application
○ Low vapor heat capacity
○ Low viscosity
○ High thermal conductivity
** ● Miscellaneous:**
○ Satisfactory oil solubility
○ High dielectric constant of vapor
○ Low freezing point
○ Reasonable containment materials
○ Easy leak detection
○ Low cost

a Table reprinted with permission from ref 42. Copyright 1987 ASHRAE, www.ashrae.org.

**Table 2.** Fluid Parameters Varied in the Optimization Runs and Their Ranges

Parameter	range
reference fluid	propane or R-32
*T*_c_/K	305−650
*p*_c_/MPa	2.0−12.0
*ω*	0.0−0.6
*C_p_*^0^(300 K)/J·mol^−1^·K^−1^	20.8−300

**Table 3.** Low-GWP Fluids Identified in Study of McLinden et al.^72^

IUPAC name	ASHRAE designation
Hydrocarbons and Dimethyl Ether	
ethane	R-170
propene (propylene)	R-1270
propane	R-290
methoxymethane (dimethyl ether)	R-E170
cyclopropane	R-C270
**Fluorinated Alkanes (HFCs)**	
fluoromethane	R-41
difluoromethane	R-32
fluoroethane	R-161
1,1-difluoroethane	R-152a
1,1,2,2-tetrafluoroethane	R-134
**Fluorinated Alkenes (HFOs) and Alkyne**	
1,1-difluoroethene	R-1132a
fluoroethene	R-1141
1,1,2-trifluoroethene	R-1123
3,3,3-trifluoroprop-1-yne	n.a.
2,3,3,3-tetrafluoroprop-1-ene	R-1234yf
(*E*)-1,2-difluoroethene	R-1132(E)
3,3,3-trifluoroprop-1-ene	R-1243zf
1,2-difluoroprop-1-ene^a^	R-1252ye^a^
(*E*)-1,3,3,3-tetrafluoroprop-1-ene	R-1234ze(E)
(*Z*)-1,2,3,3,3-pentafluoro-1-propene	R-1225ye(Z)
1-fluoroprop-1-ene^a^	R-1261ze^a^
**Fluorinated Oxygenates**	
trifluoro(methoxy)methane	R-E143a
2,2,4,5-tetrafluoro-1,3-dioxole	n.a.
**Fluorinated Nitrogen and Sulfur Compounds**	
*N*,*N*,1,1-tetrafluoromethaneamine	n.a.
difluoromethanethiol	n.a.
trifluoromethanethiol	n.a.
**Iodine Compound**	
trifluoroiodomethane	R-13I1
**Inorganic Compounds**	
carbon dioxide	R-744
ammonia	R-717

a This fluid has cis (*Z*) and trans (*E*) isomers; the predicted values of both were the same.
