# Supplementary material for: (R)Evolution of Refrigerants
Source: J Chem Eng Data. Author manuscript; Available in PMC 2022 Jan 7. (PMC8739722; doi:10.1021/acs.jced.0c00338)
Supplement: Supp1 [file NIHMS1642330-supplement-Supp1.pdf]

SUPPORTING INFORMATION

(R)Evolution of Refrigerants

Mark O. McLinden† and Marcia L. Huber

Applied Chemicals and Materials Division, National Institute of Standards and Technology
325 Broadway, Boulder, Colorado 80305 USA

Table S1. Pure-fluid refrigerants in REFPROP version 10.0 and sources of property formulations. Values for T_{NBP} and T_c are computed from the cited EOS.

ASHRAE designation	Chemical name	CAS number	Property Sources	T_{NBP}/K	T_c/K
**CFCs**					
R-11	Trichlorofluoromethane	75-69-4	EOS: Jacobsen et al. (1992)[1]<br>σ: Mulero et al. (2012)[2]<br>λ: McLinden et al. (2000)[3]<br>η: Klein et al. (1997)[4]	296.86	471.11
R-12	Dichlorodifluoromethane	75-71-8	EOS: Marx et al. (1992)[5]<br>σ: Mulero et al. (2012)[2]<br>λ: McLinden et al. (2000)[3]<br>η: Klein et al. (1997)[4]	243.40	385.12
R-13	Chlorotrifluoromethane	75-72-9	EOS: Magee at al. (2000)[6]<br>σ: Mulero et al. (2012)[2]<br>λ: Huber et al. (2003)[7,*]<br>η: Huber et al. (2003)[7,*]	191.67	302.00
R-113	1,1,2-Trichloro-1,2,2-trifluoroethane	76-13-1	EOS: Marx et al. (1992)[5]<br>σ: Mulero et al. (2012)[2]<br>λ: Huber et al. (2003)[7,*]<br>η: Huber et al. (2003)[7,*]	320.74	487.21
R-114	1,2-Dichloro-1,1,2,2-tetrafluoroethane	76-14-2	EOS: Platzer et al. (1990)[8]<br>σ: Mulero et al. (2012)[2]<br>λ: Huber (2018)[9]<br>η: Huber (2018)[9]	276.74	418.83
R-115	Chloropentafluoroethane	76-15-3	EOS: Lemmon and Span(2015)[10]<br>σ: Mulero et al. (2012)[2]<br>λ: Huber et al. (2003)[7,*]<br>η: Huber et al. (2003)[7,*]	233.93	353.10
**HCFCs and HCCs**					
R-21	Dichlorofluoromethane	75-43-4	EOS: Platzer et al. (1990)[8]<br>σ: Mulero et al. (2012)[2]<br>λ: Huber et al. (2003)[7,*]<br>η: Huber et al. (2003)[7,*]	282.01	451.48
R-22	Chlorodifluoromethane	75-45-6	EOS: Kamei et al. (1995)[11]<br>σ: Mulero et al. (2012)[2]<br>λ: McLinden et al. (2000)[3]<br>η: Klein et al. (1997)[4]	232.34	369.30

† markm@boulder.nist.gov

ASHRAE designation	Chemical name	CAS number	Property Sources	T_{NBP}/K	T_c/K
R-40	Methyl chloride	74-87-3	EOS: Thol et al. (2014)[12]<br>σ: Mulero and Cachadiña (2014)[13]<br>λ: Huber (2018)[9]<br>η: Huber (2018)[9]	249.17	416.30
R-123	2,2-Dichloro-1,1,1-trifluoroethane	306-83-2	EOS: Younglove and McLinden (1994)[14]<br>σ: Mulero et al. (2012)[2]<br>λ: Laesecke et al. (1996)[15]<br>η: Tanaka and Sotari (1995)[16]	300.97	456.83
R-124	1-Chloro-1,2,2,2-tetrafluoroethane	2837-89-0	EOS: de Vries et al. (1995)[17]<br>σ: Mulero et al. (2012)[2]<br>λ: Huber et al. (2003)[7,*]<br>η: Huber et al. (2003)[7,*]	261.19	395.43
R-141b	1,1-Dichloro-1-fluoroethane	1717-00-6	EOS: Lemmon and Span (2006)[18]<br>σ: Mulero et al. (2012)[2]<br>λ: Huber et al. (2003)[7,*]<br>η: Huber et al. (2003)[7,*]	305.20	477.50
R-142b	1-Chloro-1,1-difluoroethane	75-68-3	EOS: Lemmon and Span (2006)[18]<br>σ: Mulero et al. (2012)[2]<br>λ: Huber et al. (2003)[7,*]<br>η: Huber et al. (2003)[7,*]	264.03	410.26
R-150	1,2-Dichloroethane	107-06-2	EOS: Thol et al. (2017)[19]<br>σ: Huber (2018)[9]<br>λ: Huber (2018)[9]<br>η: Huber (2018)[9]	356.65	561.60
**HFCs**					
R-14	Tetrafluoromethane	75-73-0	EOS: Platzer et al. (1990)[8]<br>σ: Mulero et al. (2012)[2]<br>λ: Huber et al. (2003)[7,*]<br>η: Huber et al. (2003)[7,*]	145.10	227.51
R-23	Trifluoromethane	75-46-7	EOS: Penoncello at al. (2003)[20]<br>σ: Mulero et al. (2012)[2]<br>λ: Shan et al. (2000)[21]<br>η: Shan et al. (2000)[21]	191.13	299.29
R-32	Difluoromethane	75-10-5	EOS: Tillner-Roth and Yokozeki (1997)[22]<br>σ: Mulero et al. (2012)[2]<br>λ: Huber et al. (2003)[7,*]<br>η: Huber et al. (2003)[7,*]	221.50	351.26
R-41	Fluoromethane	593-53-3	EOS: : Lemmon and Span (2006)[18]<br>σ: : Lemmon and Span (2006)[18]<br>λ: McLinden et al. (2000)[3]<br>η: Klein et al. (1997)[4]	194.84	317.28
R-116	Hexafluoroethane	76-16-4	EOS: Lemmon and Span (2006)[18]<br>σ: Mulero et al. (2012)[2]<br>λ: Huber et al. (2003)[7,*]<br>η: Huber et al. (2003)[7,*]	195.06	293.03
R-125	Pentafluoroethane	354-33-6	EOS: Lemmon and Jacobsen (2005)[23]<br>σ: Mulero et al. (2012)[2]<br>λ: Perkins and Huber (2006)[24]<br>η: Huber and Laesecke (2006)[25]	225.06	339.17

ASHRAE designation	Chemical name	CAS number	Property Sources	T_{NBP}/K	T_c/K
R-134a	1,1,1,2-Tetrafluoroethane	811-97-2	EOS: Tillner-Roth and Baehr (1994)[26]<br>σ: Mulero et al. (2012)[2]<br>λ: Huber et al. (2003)[7]<br>η: Perkins et al. (2000)[27]	247.08	374.21
R-143a	1,1,1-Trifluoroethane	420-46-2	EOS: Lemmon and Jacobsen (2000)[28]<br>σ: Mulero and Cachadiña (2014)[13]<br>λ: Huber (2018)[9]<br>η: Huber (2018)[9]	225.91	345.86
R-152a	1,1-Difluoroethane	75-37-6	EOS: Outcalt and McLinden (1996)[29]<br>σ: Mulero et al. (2012)[2]<br>λ: Krauss et al. (1996)[30]<br>η: Klein et al. (2000)[4]	249.13	386.41
R-161	Fluoroethane	353-36-6	EOS: Qi et al. (2016)[31]<br>σ: Mulero et al. (2012)[2]<br>λ: Tsolakidou et al. (2017)[32]<br>η: Tsolakidou et al. (2017)[32]	235.61	375.25
R-218	Octafluoropropane	76-19-7	EOS: Lemmon and Span (2006)[18]<br>σ: Mulero et al. (2012)[2]<br>λ: Huber (2018)[9]<br>η: Huber (2018)[9]	236.36	345.02
R-227ea	1,1,1,2,3,3,3-Heptafluoropropane	431-89-0	EOS: Lemmon and Span (2015)[10]<br>σ: Mulero et al. (2012)[2]<br>λ: Huber et al. (2003)[7,*]<br>η: Huber et al. (2003)[7,*]	256.81	374.90
R-236ea	1,1,1,2,3,3-Hexafluoropropane	431-63-0	EOS: Rui et al. (2013)[33]<br>σ: Mulero et al. (2012)[2]<br>λ: Huber (2018)[9]<br>η: Huber (2018)[9]	279.32	412.44
R-236fa	1,1,1,3,3,3-Hexafluoropropane	690-39-1	EOS: Pan et al. (2012)[34]<br>σ: Mulero et al. (2012)[2]<br>λ: Huber (2018)[9]<br>η: Huber (2018)[9]	271.66	398.07
R-245ca	1,1,2,2,3-Pentafluoropropane	679-86-7	EOS: Zhou and Lemmon (2016)[35]<br>σ: Mulero et al. (2012)[2]<br>λ: Huber (2018)[9]<br>η: Huber (2018)[9]	298.41	447.57
R-245fa	1,1,1,3,3-Pentafluoropropane	460-73-1	EOS: Akasaka et al. (2015)[36]<br>σ: Mulero et al. (2012)[2]<br>λ: Perkins et al. (2016)[37]<br>η: Perkins et al. (2016)[37]	288.20	427.01
R-365mfc	1,1,1,3,3-Pentafluorobutane	406-58-6	EOS: Lemmon and Span (2015)[10]<br>σ: Mulero et al. (2012)[2]<br>λ: Huber (2018)[9]<br>η: Huber (2018)[9]	313.34	460.00
R-C318	Octafluorocyclobutane	115-25-3	EOS: Platzer et al. (1990)[8]<br>σ: Mulero et al. (2012)[2]<br>λ: Huber (2018)[9]<br>η: Huber (2018)[9]	267.18	388.38

ASHRAE designation	Chemical name	CAS number	Property Sources	T_{NBP}/K	T_c/K
**HFOs**					
R-1123	Trifluoroethene	359-11-5	EOS: Akasaka et al. (2016)[38]<br>σ: Huber (2018)[9]<br>λ: Huber (2018)[9]<br>η: Huber (2018)[9]	214.06	331.73
R-1140	Chloroethylene<br>(vinyl chloride)	75-01-4	EOS: Thol (2014)[39]<br>σ: Huber (2018)[9]<br>λ: Huber (2018)[9]<br>η: Huber (2018)[9]	259.44	424.96
R-1216	Hexafluoropropene	116-15-4	EOS: Zhou (2018)[40]<br>σ: Mulero and Cachadiña (2014)[13]<br>λ: Huber et al. (2003)[7,*]<br>η: Huber et al. (2003)[7,*]	242.81	358.90
R-1224yd(Z)	(*Z*)-1-Chloro-2,3,3,3-tetrafluoropropene	111512-60-8	EOS: Akasaka et al. (2017)[41]<br>σ: Huber (2018)[9]<br>λ: Huber (2018)[9]<br>η: Huber (2018)[9]	287.77	428.69
R-1233zd(E)	*trans*-1-Chloro-3,3,3-trifluoro-1-propene	102687-65-0	EOS: Mondejar et al. (2015)[42]<br>σ: Kondou et al.(2015)[43]<br>λ: Perkins et al. (2017)[44]<br>η: Huber (2018)[9]	291.41	439.60
R-1234yf	2,3,3,3-Tetrafluoroprop-1-ene	754-12-1	EOS: Richter et al. (2011)[45]<br>σ: Mulero et al. (2012)[2]<br>λ: Perkins and Huber (2011)[46]<br>η: Huber and Assael (2016)[47]	243.67	367.85
R-1234ze(E)	*trans*-1,3,3,3-Tetrafluoropropene	29118-24-9	EOS: Thol and Lemmon (2016)[48]<br>σ: Mulero and Cachadiña (2014)[13]<br>λ: Perkins and Huber (2011)[46]<br>η: Huber and Assael (2016)[47]	254.18	382.51
R-1234ze(Z)	*cis*-1,3,3,3-Tetrafluoropropene	29118-25-0	EOS: Akasaka and Lemmon (2019)[49]<br>σ: Kondou et al. (2015)[43]<br>λ: Huber (2018)[9]<br>η: Huber (2018)[9]	282.88	423.27
R-1243zf	3,3,3-Trifluoropropene	677-21-4	EOS: Akasaka and Lemmon (2019)[49]<br>σ: Kondou et al. (2015)[43]<br>λ: Huber (2018)[9]<br>η: Huber (2018)[9]	247.73	376.93
R-1336mzz(Z)	(*Z*)-1,1,1,4,4,4-Hexafluoro-2-butene	692-49-9	EOS: McLinden and Akasaka (2020)[50]<br>σ: Huber (2018)[9]<br>λ: Huber (2018)[9],**<br>η: Huber (2018)[9]	306.60	444.50

ASHRAE designation	Chemical name	CAS number	Property Sources	T_{NBP}/K	T_c/K
**hydrocarbons**					
R-170	Ethane	74-84-0	EOS: Bücker and Wagner (2006)[51]<br>σ: Mulero et al. (2012)[2]<br>λ: Friend et al.(1991)[52]<br>η: Vogel et al.(2015)[53]	184.57	305.32
R-290	Propane	74-98-6	EOS: Lemmon et al. (2009)[54]<br>σ: Mulero et al. (2012)[2]<br>λ: Marsh et al. (2002)[55]<br>η: Vogel and Herrmann (2016)[56]	231.04	369.89
R-600	*n*-Butane	106-97-8	EOS: Bücker and Wagner (2006)[57]<br>σ: Mulero et al. (2012)[2]<br>λ: Perkins et al. (2002)[58]<br>η: Herrmann and Vogel (2018)[59]	272.66	425.13
R-600a	2-Methylpropane (isobutane)	75-28-5	EOS: Bücker and Wagner (2006)[57]<br>σ: Mulero et al. (2012)[2]<br>λ: Perkins (2002)[60]<br>η: Vogel et al. (2000)[61]	261.40	407.81
R-601	*n*-Pentane	109-66-0	EOS: Thol (2018)[62]<br>σ: Mulero et al. (2012)[2]<br>λ: Vassiliou et al. (2015)[63]<br>η: Huber (2018)[9]	309.21	469.70
R-1150	Ethene (ethylene)	74-85-1	EOS: Smukala et al. (2000)[64]<br>σ: Mulero et al. (2012)[2]<br>λ: Assael et al. (2016)[65]<br>η: Holland et al. (1983)[66]	169.38	282.35
R-1270	Propene (propylene)	115-07-1	EOS: Lemmon (2018)[67]<br>σ: Mulero et al. (2012)[2]<br>λ: Assael et al. (2016)[65]<br>η: Huber (2018)[9]	225.53	364.21
**Ethers**					
R-E143a	Methyl trifluoromethyl ether	421-14-7	EOS: Akasaka and Kayukawa (2012)[68]<br>σ: Huber (2018)[9]<br>λ: Huber (2018)[9]<br>η: Huber (2018)[9]	249.57	377.92
R-E170	Methoxymethane (dimethyl ether)	115-10-6	EOS: Wu et al. (2011)[69]<br>σ: Mulero et al. (2012)[2]<br>λ: Huber (2018)[9]<br>η: Meng et al. (2012)[70]	248.37	400.38
R-E245cb2	Methyl pentafluoroethyl-ether	22410-44-2	EOS: Zhou (2018)[40]<br>σ: Mulero and Cachadiña (2014)[13]<br>λ: Huber (2018)[9]<br>η: Huber (2018)[9]	278.76	406.81
R-E245fa2	2,2,2-Trifluoroethyl-difluoromethyl-ether	1885-48-9	EOS: Zhou (2018)[40]<br>σ: Mulero and Cachadiña (2014)[13]<br>λ: Huber (2018)[9]<br>η: Huber (2018)[9]	302.40	444.88
R-E347mcc	1,1,1,2,2,3,3-Heptafluoro-3-methoxypropane	375-03-1	EOS: Zhou (2018)[40]<br>σ: Mulero and Cachadiña (2014)[13]<br>λ: Huber (2018)[9]<br>η: Huber (2018)[9]	307.33	437.70

ASHRAE designation	Chemical name	CAS number	Property Sources	T_{NBP}/K	T_c/K
n.a.	1,1,1,2,2,4,5,5,5-Nonafluoro-4-(trifluoromethyl)-3-pentanone (Novec 649)	756-13-8	EOS: McLinden et al. (2015)[71]<br>σ: Cui et al. (2018)[72]<br>λ: Perkins et al. (2018)[73]<br>η: Wen et al. (2017)[74]	322.20	441.81
**other**					
R-13I1	Trifluoroiodomethane	2314-97-8	EOS: Lemmon and Span (2015)[10]<br>σ: Mulero et al. (2012)[2]<br>λ: Huber (2018)[9]<br>η: Huber (2018)[9]	251.29	396.44
R-717	Ammonia	7664-41-7	EOS: Gao (2018) [75]<br>σ: Mulero et al. (2012)[2]<br>λ: Monogenidou et al. (2018)[76]<br>η: Monogenidou et al. (2018)[77]	239.83	405.56
R-718	Water	7732-18-5	EOS: Wagner and Pruß (2002)[78]<br>σ: IAPWS (2014)[79]<br>λ: Huber et al. (2012)[80]<br>η: Huber et al. (2009)[81]	373.12	647.10
R-744	Carbon dioxide	124-38-9	EOS: Span and Wagner (1996)[82]<br>σ: Mulero et al. (2012)[2]<br>λ: Huber et al. (2016)[83]<br>η: Laesecke and Muzny (2017)[84]	194.69	304.13
R-764	Sulfur dioxide	7446-09-5	EOS: Gao et al. (2016)[85]<br>σ: Mulero et al. (2012)[2]<br>λ: Huber (2018)[9]<br>η: Huber (2018)[9]	263.14	430.64

*Coefficients are in Ref.[86] using method described in Ref.[7] **Now superceded by Ref.[87]

REFERENCES

1. Jacobsen, R. T.; Penoncello, S. G.; Lemmon, E. W., A fundamental equation for trichlorofluoromethane (R-11). *Fluid Phase Equilib.* **1992,** *80*, 45-56.
2. Mulero, A.; Cachadiña, I.; Parra, M. I., Recommended correlations for the surface tension of common fluids. *J. Phys. Chem. Ref. Data* **2012,** *41*, 043105.
3. McLinden, M. O.; Klein, S. A.; Perkins, R. A., An extended corresponding states model for the thermal conductivity of refrigerants and refrigerant mixtures. *Int. J. Refrig.* **2000,** *23*, 43-63.
4. Klein, S. A.; McLinden, M. O.; Laesecke, A., An improved extended corresponding states method for the estimation of viscosity of pure refrigerants and mixtures. *Int. J. Refrig.* **1997,** *20*, 208-217.
5. Marx, V.; Pruss, A.; Wagner, W., Neue Zustandsgleichungen für R 12, R 22, R 11 und R 113. Beschreibung des thermodynamishchen Zustandsverhaltens bei Temperaturen bis 525 K und Druecken bis 200 MPa, Duesseldorf, VDI Verlag, Series 19 (Waermetechnik/ Kaeltetechnik), No. 57, 1992.
6. Magee, J. W.; Outcalt, S. L.; Ely, J. F., Molar heat capacity C_v, vapor pressure, and (p, ρ, T) measurements from 92 to 350 K at pressures to 35 MPa and a new equation of state for chlorotrifluoromethane (R13). *Int. J. Thermophys.* **2000,** *21*, 1097-1121.
7. Huber, M. L.; Laesecke, A.; Perkins, R. A., Model for the viscosity and thermal conductivity of refrigerants, including a new correlation for the viscosity of R134a. *Ind. Eng. Chem. Res.* **2003,** *42*, 3163-3178.
8. Platzer, B.; Polt, A.; Maurer, G., *Thermophysical Properties of Refrigerants*. Springer-Verlag: Berlin, 1990.
9. Huber, M. L., Models for the viscosity, thermal conductivity, and surface tension of selected pure fluids as implemented in REFPROP v10.0, NISTIR 8209, 2018. doi: 10.6028/NIST.IR.8209.
10. Lemmon, E. W.; Span, R., Thermodynamic properties of R-227ea, R-365mfc, R-115, and R-13I1. *J. Chem. Eng. Data* **2015,** *60*, 3745-3758.
11. Kamei, A.; Beyerlein, S. W.; Jacobsen, R. T., Application of nonlinear regression in the development of a wide range formulation for HCFC-22. *Int. J. Thermophys.* **1995,** *16*, 1155-1164.
12. Thol, M.; Piazza, L.; Span, R., A new functional form for equations of state for some weakly associating fluids. *Int. J. Thermophys.* **2014,** *35*, 783-811.
13. Mulero, A.; Cachadiña, I., Recommended correlations for the surface tension of several fluids included in the REFPROP program. *J. Phys. Chem. Ref. Data* **2014,** *43*, 023104.
14. Younglove, B. A.; McLinden, M. O., An international standard equation of state for the thermodynamic properties of refrigerant 123 (2,2-Dichloro-1,1,1-Trifluoroethane). *J. Phys. Chem. Ref. Data* **1994,** *23*, 731-779.
15. Laesecke, A.; Perkins, R. A.; Howley, J. B., An improved correlation for the thermal conductivity of HCFC123 (2,2-dichloro-1,1,1-trifluoroethane). *Int. J. Refrig.* **1996,** *19*, 231-238.
16. Tanaka, Y.; Sotani, T., Thermal conductivity and viscosity of 2,2-dichloro-1,1,1-trifluoroethane (HCFC-123). *Int. J. Thermophys.* **1996,** *17*, 293-328.

17. de Vries, B.; Tillner-Roth, R.; Baehr, H. D., Thermodynamic properties of HCFC 124, 19th International Congress of Refrigeration, The Hague, The Netherlands, International Institute of Refrigeration, IVa:582-589, 1995.

18. Lemmon, E. W.; Span, R., Short fundamental equations of state for 20 industrial fluids. *J. Chem. Eng. Data* **2006,** *51*, 785-850.

19. Thol, M.; Koeste, A.; Rutkai, G.; Span, R.; Wagner, W.; Vrabec, J., Equation of state for dichloroethane based on a hybrid data set. *Mol. Phys.* **2017,** *115*, 1166-1185.

20. Penoncello, S. G.; Lemmon, E. W.; Jacobsen, R. T.; Shan, Z. J., A fundamental equation for trifluoromethane (R-23). *J. Phys. Chem. Ref. Data* **2003,** *32*, 1473-1499.

21. Shan, Z.; Penoncello, S. G.; Jacobsen, R. T., A generalized model for viscosity and thermal conductivity of trifluoromethane (R-23). *ASHRAE Trans.* **2000,** *106*, 757-767.

22. Tillner-Roth, R.; Yokozeki, A., An international standard equation of state for difluoromethane (R-32) for temperatures from the triple point at 136.34 K to 435 K and pressures up to 70 MPa. *J. Phys. Chem. Ref. Data* **1997,** *26*, 1273-1328.

23. Lemmon, E. W.; Jacobsen, R. T., A new functional form and new fitting techniques for equations of state with application to pentafluoroethane (HFC-125). *J. Phys. Chem. Ref. Data* **2005,** *34*, 69-108.

24. Perkins, R. A.; Huber, M. L., Measurement and correlation of the thermal conductivity of pentafluoroethane (R125) from 190 K to 512 K at pressures to 70 MPa. *J. Chem. Eng. Data* **2006,** *51*, 898-904.

25. Huber, M. L.; Laesecke, A., Correlation for the viscosity of pentafluoroethane (R125) from the triple point to 500 K at pressures up to 60 MPa. *Ind. Eng. Chem. Res.* **2006,** *45*, 4447-4453.

26. Tillner-Roth, R.; Baehr, H. D., An international standard formulation of the thermodynamic properties of 1,1,1,2-tetrafluoroethane (HFC-134a) for temperatures from 170 K to 455 K at pressures up to 70 MPa. *J. Phys. Chem. Ref. Data* **1994,** *23*, 657-729.

27. Perkins, R. A.; Laesecke, A.; Howley, J. B.; Ramires, M. L. V.; Gurova, A. N.; Cusco, L., Experimental thermal conductivity values for the IUPAC round-robin sample of 1,1,1,2-tetrafluoroethane (R134a), NISTIR, 2000.

28. Lemmon, E. W.; Jacobsen, R. T., An international standard formulation for the thermodynamic properties of 1,1,1-trifluoroethane (HFC-143a) for temperatures from 161 to 450 K and pressures to 50 MPa. *J. Phys. Chem. Ref. Data* **2000,** *29*, 521-552.

29. Outcalt, S. L.; McLinden, M. O., A modified Benedict-Webb-Rubin equation of state for the thermodynamic properties of R152a (1,1-difluoroethane). *J. Phys. Chem. Ref. Data* **1996,** *25*, 605-636.

30. Krauss, R.; Weiss, V. C.; Edison, T. A.; Sengers, J. V.; Stephan, K., Transport properties of 1,1-difluoroethane (R152a). *Int. J. Thermophys.* **1996,** *17*, 731-757.

31. Qi, H. Y.; Fang, D.; Gao, K. H.; Meng, X. Y.; Wu, J. T., Compressed liquid densities and Helmholtz energy equation of state for fluoroethane (R161). *Int. J. Thermophys.* **2016,** *37*, 55.

32. Tsolakidou, C. M.; Assael, M. J.; Huber, M. L.; Perkins, R. A., Correlations for the viscosity and thermal conductivity of ethyl fluoride (R161). *J. Phys. Chem. Ref. Data* **2017,** *46*, 023103.

33. Rui, X. F.; Pan, J.; Wang, Y. G., An equation of state for the thermodynamic properties of 1,1,1,2,3,3-hexafluoropropane (R236ea). *Fluid Phase Equilib.* **2013,** *341*, 75-85.

34. Pan, J.; Rui, X. F.; Zhao, X. D.; Qiu, L. M., An equation of state for the thermodynamic properties of 1,1,1,3,3,3-hexafluoropropane (HFC-236fa). *Fluid Phase Equilib.* **2012,** *321*, 10-16.

35. Zhou, Y.; Lemmon, E. W., Equation of state for the thermodynamic properties of 1,1,2,2,3-pentafluoropropane (R-245ca). *Int. J. Thermophys.* **2016,** *37*, 27.

36. Akasaka, R.; Zhou, Y.; Lemmon, E. W., A fundamental equation of state for 1,1,1,3,3-pentafluoropropane (R-245fa). *J. Phys. Chem. Ref. Data* **2015,** *44*, 013104.

37. Perkins, R. A.; Huber, M. L.; Assael, M. J., Measurements of the thermal conductivity of 1,1,1,3,3-Pentafluoropropane (R245fa) and correlations for the viscosity and thermal conductivity surfaces. *J. Chem. Eng. Data* **2016,** *61*, 3286-3294.

38. Akasaka, R.; Fukushima, M.; Lemmon, E. W., A Helmholtz energy equation of state for trifluoroethylene (R-1123), International Refrigeration and Air Conditioning Conference at Purdue, July 11-14, 2016.

39. Thol, M., Ruhr-Univ. Bochum, Germany, Helmholtz equation of state for vinyl chloride. **2014**.

40. Zhou, Y., Xi'an Xioatong Univ., China, Equations of state for RE245cb2, RE347mcc, RE245fa2, and R1216. **2018**.

41. Akasaka, R.; Fukushima, M.; Lemmon, E. W., A Helmholtz energy equation of state for cis-1-chloro-2,3,3,3-tetrafluoropropene (R-1224yd(Z)), European Conference on Thermophysical Properties, Graz, Austria, September 3-8, 2017.

42. Mondejar, M. E.; McLinden, M. O.; Lemmon, E. W., Thermodynamic properties of *trans*-1-chloro-3,3,3-trifluoropropene (R1233zd(E)): Vapor pressure, (p, ρ, T) behavior, and speed of sound measurements, and equation of state. *J. Chem. Eng. Data* **2015,** *60*, 2477-2489.

43. Kondou, C.; Nagata, R.; Nii, N.; Koyama, S.; Higashi, Y., Surface tension of low GWP refrigerants R1243zf, R1234ze(Z), and R1233zd(E). *Int. J. Refrig.* **2015,** *53*, 80-89.

44. Perkins, R. A.; Huber, M. L.; Assael, M. J., Measurement and correlation of the thermal conductivity of trans-1-chloro-3,3,3-trifluoropropene (R1233zd(E)). *J. Chem. Eng. Data* **2017,** *62*, 2659-2665.

45. Richter, M.; McLinden, M. O.; Lemmon, E. W., Thermodynamic properties of 2,3,3,3-tetrafluoroprop-1-ene (R1234yf): Vapor pressure and p–ρ–T measurements and an equation of state. *J. Chem. Eng. Data* **2011,** *56*, 3254-3264.

46. Perkins, R. A.; Huber, M. L., Measurement and correlation of the thermal conductivity of 2,3,3,3-tetrafluoroprop-1-ene (R1234yf) and trans-1,3,3,3-tetrafluoropropene (R1234ze(E)). *J. Chem. Eng. Data* **2011,** *56*, 4868-4874.

47. Huber, M. L.; Assael, M. J., Correlations for the viscosity of 2,3,3,3-tetrafluoroprop-1-ene (R1234yf) and trans-1,3,3,3-tetrafluoropropene (R1234ze(E)). *Int. J. Refrig.* **2016,** *71*, 39-45.

48. Thol, M.; Lemmon, E. W., Equation of state for the thermodynamic properties of trans-1,3,3,3-tetrafluoropropene R-1234ze(E). *Int. J. Thermophys.* **2016,** *37*, 28.

49. Akasaka, R.; Lemmon, E. W., Fundamental equations of state for cis-1,3,3,3-tetrafluoropropene R-1234ze(Z) and 3,3,3-trifluoropropene (R-1243zf). *J. Chem. Eng. Data* **2019,** *64*, 4679-4691.

50. McLinden, M. O.; Akasaka, R., Thermodynamic properties of cis-1,1,1,4,4,4-hexafluorobutene [R-1336mzz(Z)]: Vapor pressure, (p, ρ, T) behavior, and speed of sound measurements and equation of state. *J. Chem. Eng. Data* **2020** (in press, doi 10.1021/acs.jced.9b01198).

51. Bücker, D.; Wagner, W., A reference equation of state for the thermodynamic properties of ethane for temperatures from the melting line to 675 K and pressures up to 900 MPa. *J. Phys. Chem. Ref. Data* **2006,** *35*, 205-266.

52. Friend, D. G.; Ingham, H.; Ely, J. F., Thermophysical properties of ethane. *J. Phys. Chem. Ref. Data* **1991,** *20*, 275-347.

53. Vogel, E.; Span, R.; Herrmann, S., Reference correlation for the viscosity of ethane. *J. Phys. Chem. Ref. Data* **2015,** *44*, 043101.

54. Lemmon, E. W.; McLinden, M. O.; Wagner, W., Thermodynamic properties of propane. III. A reference equation of state for temperatures from the melting line to 650 K and pressures up to 1000 MPa. *J. Chem. Eng. Data* **2009,** *54*, 3141-3180.

55. Marsh, K. N.; Perkins, R. A.; Ramires, M. L. V., Measurement and correlation of the thermal conductivity of propane. *J. Chem. Eng. Data* **2002,** *47*, 932-940.

56. Vogel, E.; Herrmann, S., New formulation for the viscosity of propane. *J. Phys. Chem. Ref. Data* **2016,** *45*, 043103.

57. Bücker, D.; Wagner, W., Reference equations of state for the thermodynamic properties of fluid phase n-butane and isobutane. *J. Phys. Chem. Ref. Data* **2006,** *35*, 929-1019.

58. Perkins, R. A.; Ramires, M. L. V.; Nieto de Castro, C. A.; Cusco, L., Measurement and correlation of the thermal conductivity of butane from 135 K to 600 K at pressures to 70 MPa. *J. Chem. Eng. Data* **2002,** *47*, 1263-1271.

59. Herrmann, S.; Vogel, E., New formulation for the viscosity of n-butane. *J. Phys. Chem. Ref. Data* **2018,** *47*, 013104.

60. Perkins, R. A., Measurement and correlation of the thermal conductivity of isobutane from 114 K to 600 K at pressures to 70 MPa. *J. Chem. Eng. Data* **2002,** *47*, 1272-1279.

61. Vogel, E.; Kuchenmeister, C.; Bich, E., Viscosity correlation for isobutane over wide ranges of the fluid region. *Int. J. Thermophys.* **2000,** *21*, 343-356.

62. Thol, M., Ruhr-Univ. Bochum, Germany, Fundamental equations of state for hydrocarbons. Part I. n-Pentane. 2018.

63. Vassiliou, C. M.; Assael, M. J.; Huber, M. L.; Perkins, R. A., Reference correlations of the thermal conductivity of cyclopentane, iso-pentane, and n-pentane. *J. Phys. Chem. Ref. Data* **2015,** *44,* 033102.

64. Smukula, J.; Span, R.; Wagner, W., A new equation of state for ethylene covering the fluid region for temperatures from the melting line to 450 K at pressures up to 300 MPa. *J. Phys. Chem. Ref. Data* **2000,** *29*, 1052-1122.

65. Assael, M. J.; Koutian, A.; Huber, M. L.; Perkins, R. A., Reference correlations of the thermal conductivity of ethene and propene. *J. Phys. Chem. Ref. Data* **2016,** *45*, 033104.

66. Holland, P. M.; Eaton, B. E.; Hanley, H. J. M., A correlation of the viscosity and thermal conductivity data of gaseous and liquid Ethylene. *J. Phys. Chem. Ref. Data* **1983,** *12*, 917-932.

67. Lemmon, E. W., NIST, Boulder, USA, A reference equation of state for propylene for temperatures from the melting line to 575 K and pressures up to 1000 MPa. 2018.

68. Akasaka, R.; Kayukawa, Y., A fundamental equation of state for trifluoromethyl methyl ether (HFE-143m) and its application to refrigeration cycle analysis. *Int. J. Refrig.* **2012,** *35*, 1003-1013.

69. Wu, J. T.; Zhou, Y.; Lemmon, E. W., An equation of state for the thermodynamic properties of dimethyl ether. *J. Phys. Chem. Ref. Data* **2011,** *40*, 023104.

70. Meng, X. Y.; Zhang, J. B.; Wu, J. T.; Liu, Z. G., Experimental measurement and modeling of the viscosity of dimethyl ether. *J. Chem. Eng. Data* **2012,** *57*, 988-993.

71. McLinden, M. O.; Perkins, R. A.; Lemmon, E. W.; Fortin, T. J., Thermodynamic properties of 1,1,1,2,2,4,5,5,5-nonafluoro-4-(trifluoromethyl)-3-pentanone: Vapor pressure, (p, ρ, T) behavior, and speed of sound measurements, and an equation of state. *J. Chem. Eng. Data* **2015,** *60*, 3646-3659.

72. Cui, J. W.; Yan, S. M.; Bi, S. S.; Wu, J. T., Saturated liquid dynamic viscosity and surface tension of trans-1-chloro-3,3,3-trifluoropropene and dodecafluoro-2-methylpentan-3-one. *J. Chem. Eng. Data* **2018,** *63*, 751-756.

73. Perkins, R. A.; Huber, M. L.; Assael, M. J., Measurement and correlation of the thermal conductivity of 1,1,1,2,2,4,5,5,5-nonafluoro-4-(trifluoromethyl)-3-pentanone. *J. Chem. Eng. Data* **2018,** *63*, 2783-2789.

74. Wen, C. Y.; Meng, X. Y.; Huber, M. L.; Wu, J. T., Measurement and correlation of the viscosity of 1,1,1,2,2,4,5,5,5-nonafluoro-4-(trifluoromethyl)-3-pentanone. *J. Chem. Eng. Data* **2017,** *62*, 3603-3609.

75. Gao, K. H., Xi'an Xiaotong Univ., China, Thermodynamic properties of ammonia for temperatures from the melting line to 725 K and pressures to 1000 MPa. 2018.

76. Monogenidou, S. A.; Assael, M. J.; Huber, M. L., Reference correlation for the thermal conductivity of ammonia from the triple-point temperature to 680 K and pressures up to 80 MPa. *J. Phys. Chem. Ref. Data* **2018,** *47*, 023102.

77. Monogenidou, S. A.; Assael, M. J.; Huber, M. L., Reference correlation for the viscosity of ammonia from the triple point to 725 K and up to 50 MPa. *J. Phys. Chem. Ref. Data* **2018,** *47*, 043101.

78. Wagner, W.; Pruß, A., The IAPWS formulation 1995 for the thermodynamic properties of ordinary water substance for general and scientific use. *J. Phys. Chem. Ref. Data* **2002,** *31*, 387-535.

79. IAPWS, International Association for the Properties of Water and Steam, Revised Release on Surface Tension of Ordinary Water Substance, IAPWS R1-76(2014), June 2014. http://www.iapws.org/relguide/Surf-H2O.html. 2014.

80. Huber, M. L.; Perkins, R. A.; Friend, D. G.; Sengers, J. V.; Assael, M. J.; Metaxa, I. N.; Miyagawa, K.; Hellmann, R.; Vogel, E., New international formulation for the thermal conductivity of H_2O. *J. Phys. Chem. Ref. Data* **2012,** *41,* 033102.

81. Huber, M. L.; Perkins, R. A.; Laesecke, A.; Friend, D. G.; Sengers, J. V.; Assael, M. J.; Metaxa, I. N.; Vogel, E.; Mareš, R.; Miyagawa, K., New international formulation for the viscosity of H_2O. *J. Phys. Chem. Ref. Data* **2009,** *38*, 101-125.

82. Span, R.; Wagner, W., A new equation of state for carbon dioxide covering the fluid region from the triple-point temperature to 1100 K at pressures up to 800 MPa. *J. Phys. Chem. Ref. Data* **1996,** *25*, 1509-1596.

83. Huber, M. L.; Sykioti, E. A.; Assael, M. J.; Perkins, R. A., Reference correlation of the thermal conductivity of carbon dioxide from the triple point to 1100 K and up to 200 MPa. *J. Phys. Chem. Ref. Data* **2016,** *45*, 013102.

84. Laesecke, A.; Muzny, C. D., Reference correlation for the viscosity of carbon dioxide. *J. Phys. Chem. Ref. Data* **2017,** *46*, 013107.

85. Gao, K. H.; Wu, J. T.; Zhang, P. G.; Lemmon, E. W., A Helmholtz energy equation of state for sulfur dioxide. *J. Chem. Eng. Data* **2016,** *61*, 2859-2872.

86. Lemmon, E. W.; Bell, I. H.; Huber, M. L.; McLinden, M. O. *NIST Standard Reference Database 23, NIST Reference Fluid Thermodynamic and Transport Properties, version 10.0*, Standard Reference Data Program, National Institute of Standards and Technology, Gaithersburg, MD, https://www.nist.gov/srd/refprop, 2018

87. Perkins, R.A.; Huber, M.L., Measurement and correlation of the thermal conductivity of cis-1,1,1,4,4,4-hexafluoro-2-butene. *Int. J. Thermophys.* **2020**, 41, 103.
